# SENP3 promotes renal tubular epithelial cell apoptosis after ischemia-reperfusion injury via ASS1 deSUMOylation

**DOI:** 10.1038/s41419-025-08308-2

**Published:** 2025-12-05

**Authors:** Hongju Wang, Cui Gao, Jingjuan Yang, Lini Jin, Longlong Wu, Qian Zhang, Xin Fang, Hong Pan, Huijuan Wu, Pattarin Tangtanatakul, Weiqiang Lin, Yi Yang

**Affiliations:** 1 Department of Nephrology, Center for Regeneration and Aging Medicine, the Fourth Affiliated Hospital of School of Medicine, and International School of Medicine, International Institutes of Medicine, Zhejiang University, Yiwu, China; 2 Zhejiang-Denmark Joint Laboratory of Regeneration and Aging Medicine, Yiwu, China; 3 Department of Nephrology, Jining No.1 People’s Hospital, Jining, China; 4 Department of Pathology, School of Basic Medical Sciences, Fudan University, Shanghai, China; 5 Centre of Excellent in Immunology and Immune-Mediated Diseases, Department of Microbiology, Chulalongkorn University, Bangkok, Thailand

**Keywords:** Acute kidney injury, Apoptosis

## Abstract

The balance between SUMOylation and deSUMOylation critically regulate cellular apoptosis, with SUMO-modified proteins implicated in ischemia/hypoxia injury. However, the specific contributions of SUMO-conjugated proteins in renal ischemia-reperfusion injury (IRI) remain poorly defined. SUMOylation in IRI was investigated Using proximal tubular-specific *Senp3* conditional knockout (CKO) mice. While SENP3-deficiency did not induce tubular injury under basal conditions, its significantly attenuated renal damage following IRI. SUMOylation conferred protection against apoptosis in renal tubular epithelia cells during ischemia/hypoxia. Mass spectrometry revealed arginosuccinate synthase 1 (ASS1) as a key SUMO2/3 target (modified at K239 and K310) in IRI progression. Mechanistically, SENP3-mediated deSUMOylation promoted ASS1 nuclear accumulation in post-IRI tubular epithelial cells, subsequently activating the intrinsic apoptosis pathway via p53-dependent transcriptional upregulation. These findings nominate the SENP3-ASS1-p53 axis as a potential therapeutic target for renal IRI.

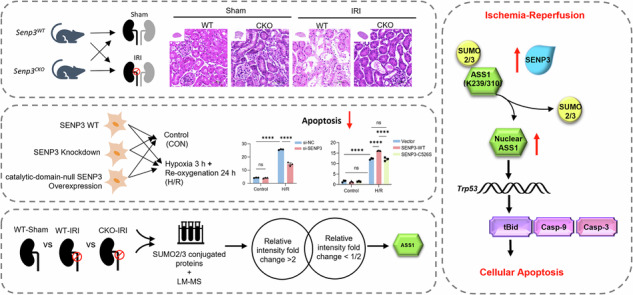

## Introduction

SUMOylation, a reversible post-translation modification, entails the covalent conjugation of small ubiquitin-like modifier (SUMO) proteins to lysine residues on target proteins. This process is dynamically regulated by SUMO-specific proteases (SENPs) [1]. Among SENPs, SENP3 localizes predominantly to the nuclear and selectively regulates both SUMO2/3 maturation and deconjugation. Notably, SENP3 exhibits redox-sensitive, oxidative stress triggers its redistribution from nucleoli to the nucleoplasm by inhibiting degradation [2–4]. SUMOylation primarily modulates nuclear process, including gene expression, genome stability, RNA processing, nucleocytoplasmic transport and cell cycle progression [5]. The equilibrium between SUMOylation and deSUMOylation critically influences cell fate, with outcomes contingent on disease context, SUMO isoforms specificity, substrate identity, and cell type. Apoptosis, among other cell death modalities, has been extensively linked to SUMOylation [6, 7].

As evolutionarily conserved regulators essential for eukaryotic cell viability, SUMO family members are frequently dysregulated in human diseases. SUMOylation can alter target protein activity, stability or subcellular localization, often by modifying interactions with macromolecules (e.g., proteins, DNA, or RNA) [8–10]. For instance, SUMOylation of Sirt1 and Eef2 in cardiomyocytes [11], Drp1 in hepatocytes [12], and Nrf2 in neuronal [13] mitigates apoptosis induced by ischemia-reperfusion injury (IRI).

In kidney diseases, SUMOylation participates in acute kidney injury (AKI), diabetic nephropathy, fibrosis and renal cell carcinoma, though mechanistic details remain debated [14]. IRI-associated AKI features proximal tubular epithelial cell detachment, dysfunction and apoptosis, a major driver of renal functional decline [15, 16]. Prior studies suggested that SUMOylation may exert cytoprotective effects in renal tubular cells [17–19]; however, the precise mechanisms underlying SUMOylation in renal IRI remain unclear. Moreover, the repertoire of SUMOylated proteins that promote tubular epithelial cell injury has yet to be comprehensively defined. Identifying these substrates is critical for elucidating IRI pathogenesis and developing targeted therapies.

Here, we demonstrate that SENP3-mediated SUMO2/3 homeostasis is a pivotal regulatory of apoptosis in renal IRI. We identify argininosuccinate synthase (ASS1) as a key SUMO2/3-modified protein in IRI-induced apoptosis. SENP3-driven deconjugation of ASS1 promotes its nuclear accumulation, activating pro-apoptotic *Trp53* gene expression and culminating in intrinsic pathway-mediated apoptosis.

## Methods

### Animal experiments

*Senp3*^*flox/flox*^ mice (provided by Prof. Jing Yi and Prof. Jie Yang, Shanghai Jiao Tong University School of Medicine, China) were crossed with *Ggt1*^*Cre*^ mice (provided by Prof. Huijuan Wu, School of Basic Medical Sciences, Fudan University, China) to generate proximal tubule-specific *Senp3* knockout mice (*Senp3*^*flox/flox*^*-Ggt1*^*Cre*^, CKO) and littermate controls (*Senp3*^*flox/flox*^, WT). Mice were housed under specific-pathogen-free conditions (20–24 °C, 50–60% humidity, 12-h light/dark cycle). The mice were randomly divided into the sham-surgery group and the IRI-AKI group. For IRI-AKI modeling, 8–10-week-old male mice underwent right nephrectomy followed by left renal pedicle clamping (30 min) or sham surgery. Reperfusion was confirmed by observing restoration of renal blood flow and color recovery, and only successful reperfusion kidneys were included for subsequent analysis [20]. Following 24 h of reperfusion, the mice were euthanized with subsequent collection of serum and kidney samples. Throughout this collection process, the experimental group assignments of the samples were not considered. All procedures were approved by the Committees for Animal Experiments of Zhejiang University.

### Cell culture and interventions

HEK293T (RRID: CVCL_0063) and TCMK1 (RRID: CVCL_2772) cells were provided by Servicebio Technology Co., Ltd. (STCC10301G, STCC20015G), and were identified by short tandem repeat (STR) profiling. All the cells used have undergone quality testing, and there were no detections of bacteria, fungi or mycoplasma. TCMK1 cells were cultured in RPMI 1640 and HEK-293T in DMEM (10% FBS, 1% penicillin/streptomycin) at 37 °C (21% O_2_, 5% CO_2_). Hypoxia/reoxygenation (H/R) was induced in TCMK1 cells by serum starvation (overnight), hypoxia (0.1% O_2_, 3 h), and reoxygenation (21% O_2_, 24 h).

For functional studies, Lentiviral transduction was carried out using constructs expressing SENP3-WT-HIS, SENP3-C526S-HIS (catalytically inactive mutant), or empty vector (from Vigen Biotechnology). Hypoxia assays were performed 48 h after cell transfection with lentiviral expression vector in medium containing polybrene (5 μg/ml). Plasmid overexpression vectors were constructed in pcDNA3.1(+), including ASS1-WT-FLAG, ASS1-K215R-FLAG, ASS1-K228R-FLAG, ASS1-K239R-FLAG, ASS1-K310R-FLAG, ASS1-K348R-FLAG, ASS1-K239/310R-FLAG, SUMO2/3-MYC, UBC9-HA (all from GenePharma Technology). For siRNA-mediated knockdown: SENP3 siRNA (GenePharma Technology, sense: ACAGCUUCUUCUAUGAUAATT, antisense: UUAUCAUAGAAGAAGCUGUUG) or ASS1 (Sangon Biotech, sense: CUGAUGGAGUAUGCAAAGCAA, antisense: UUGCUUUGCAUACUCCAUCAG). Hypoxia treatment was initiated 24 h after transfection of overexpression plasmids or siRNAs using jetPRIME reagent (Polyplus). Pharmacological inhibition was performed with the ASS1 inhibitor α-Methyl-DL-aspartic acid (MDLA, 20 mM, 24 h; MCE, HY-W142119).

### Renal function and histopathology

Serum creatinine and BUN were measured (FUJIDRI-CHEM 7000i). Kidney sections (4 μm) were stained with Hematoxylin and Eosin (H&E) and Periodic Acid-Schiff (PAS). Tubular damage was scored (0-4) based on brush border loss, dilatation and disruption, epithelial flattened and cell sloughing.

### Immunofluorescence and TUNEL assay

PFA-fixed samples were permeabilized (0.5% Triton X-100), blocked (5% BSA), and incubated with primary antibodies (SENP3, #5591, CST; ASS1, 16210-1-AP, Proteintech; LTL, L32480, Invitrogen; P53, 60283-2-Ig, Proteintech) followed by DAPI. TUNEL staining (APO001 kit, Multi Sciences) dected apoptotic cells. Images were acquired via confocal microscope (Nikon A1 Ti/Zeiss LSM 900) and analyzed with ImageJ.

### Western Blot and Immunoprecipitation (IP)

Proteins were extracted using RIPA lysis buffer (containing 1 mM PMSF) or Pierce^TM^ IP lysis buffer (Thermo Scientific, 87787). For nuclear/cytoplasmic fractionation, cells were lysed on ice with a hypotonic solution (20 mM Tris-HCl, pH 7.4; 10 mM NaCl; 3 mM MgCl₂) for 15 min. Subsequently, 10% NP-40 was added, and the mixture was vortexed and centrifuged. The supernatant containing the cytoplasmic fraction was collected. The pellet, which contained cell nuclei, was washed three times with hypotonic solution. Nuclear proteins were then extracted by lysing the pellet with RIPA buffer.

Protein samples were separated by SDS-PAGE and were subsequently transferred onto PVDF membranes. Blots were incubated with 5% fat-free milk in TBS containing 1% Tween for 1 h and incubated with primary antibodies at 4 °C overnight. Then the membranes were incubated with horseradish peroxidase-conjugated secondary antibodies.

For IP, lysates were incubated with the indicated primary antibodies or IgG in combination with protein A/G beads (Beyotime, P2108) overnight (4 °C), washed and eluted for immunoblotting. The SUMO2/3-conjugated proteins identification was performed by LC-MS using a Thermo Fusion Lumos and analyzed by Proteome Discoverer software 2.1. The details of primary antibodies were listed in Table S1.

### qRT-PCR

Total RNA was extracted (Accurate Biology, AG21023), reverse-transcribed to cDNA with gDNA clean for qPCR (Evo M-MLV RT Mix kit, Accurate Biology, AG11728), and amplified (SYBR Green Premix PCR Master Mix, Accurate Biology, AG11701) on a CFX96 instrument (Bio-Rad). Primers were listed in Table S2.

### Flow cytometry assay

10^5^ cells were collected and centrifuged at 300 g for 5 min and the supernatant was discarded. And the pellet was resuspended in 500 μL 1×binding buffer, and later incubated with 5 μL FITC-conjugated Annexin V and 10 μL propidium iodide (PI) for 5 min at 37 °C in the dark. The samples were analyzed by a flow cytometer (BD Bioscience and Beckman).

### Luciferase reporter assay

TCMK1 cells were co-transfected with pGL3-Luc (with p53 binding cis-element) plasmid and Prl-tk Renilla luciferase plasmid before establishing hypoxia/re-oxygenation (H/R) model. The cells were collected for luciferase assay (Dual Luciferase Reporter Assay Kit, Vazyme, DL101) and normalized to the Renilla.

### Statistical analysis

All experiments were repeated at least 3 times. Data are mean ± SEM (*n* ≥ 3) and analyzed using GraphPad Prism 9.3. Data normality was assessed using the Shapiro–Wilk test, and homogeneity of variances was evaluated with Levene’s test. For comparisons between two groups meeting both assumptions, Student’s *T*-test was applied. If the normality assumption was violated, the Mann–Whitney *U* test was used; if variances were unequal, Welch’s *t*-test was employed. Multiple group comparisons were performed using one-way ANOVA (with Tukey’s post-hoc test for equal variances or Games-Howell post-hoc test for unequal variances) or two-way ANOVA with Sidak’s post-hoc test, as appropriate for the experimental design. *P* < 0.05 was defined as statistically significant.

## Results

### SENP3 promotes apoptosis in renal ischemia-reperfusion injury (IRI)

**Fig. 1:**
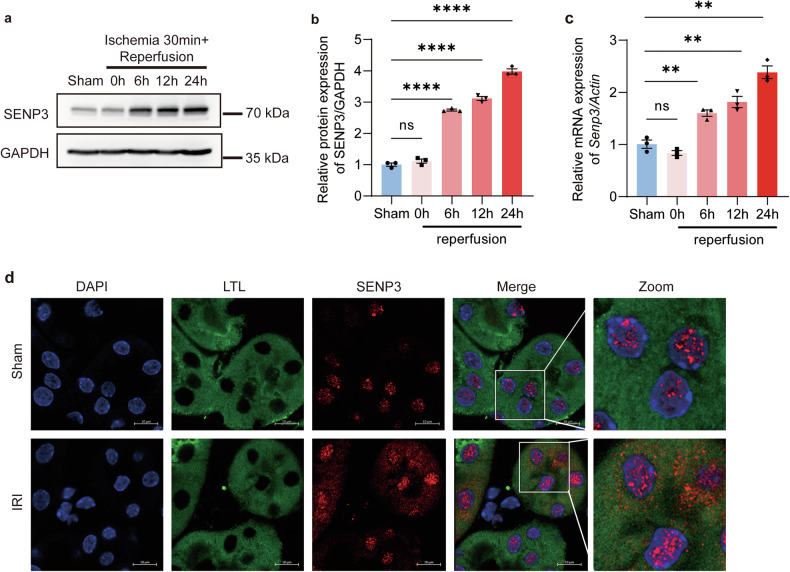
SENP3 expression is transcriptional upregulated in PTECs following IRI. **a**–**c** WT mice underwent unilateral nephrectomy followed by 30 min ischemia induced by left renal pedicle clamping and subsequent reperfusion for 6, 12, or 24 h. Both SENP3 protein expression and mRNA levels increased progressively with prolonged reperfusion time during in IRI-AKI progression. **d** Immunofluorescence staining of renal tissue showed elevated SENP3 (red) expression in PTECs, which were labeled with Lotus tetragonolobus lectin (LTL, green), at 24 h after reperfusion after reperfusion following 30 min of ischemia. Scale bar, 10 μm. Data: mean ± SEM (*n* = 3). ns, not significance. **p < 0.01. ****p < 0.0001.

We initially investigated the roles of SUMOylation and deSUMOylation in kidney ischemia-reperfusion injury (IRI). Analysis of SENP family expression in mouse kidney tissue revealed that the transcriptional levels of SENP3 and SENP5 were significantly elevated in IRI-induced acute kidney injury (IRI-AKI) mice (Fig. S1). Similar findings were observed in TCMK1 cells subjected to hypoxia/reoxygenation (H/R) (Fig. S2). Among these, SENP3 exhibited a slightly more pronounced increase than SENP5 in TCMK1 cells under H/R condition, prompting us to select SENP3 for further study. Both transcription and protein levels of SENP3 increased progressively with prolonged reperfusion time (Fig. 1a–c). Immunofluorescence staining further confirmed transcriptional upregulated SENP3 expression in proximal tubular epithelia cells (PTECs) following IRI (Fig. 1d).

**Fig. 2:**
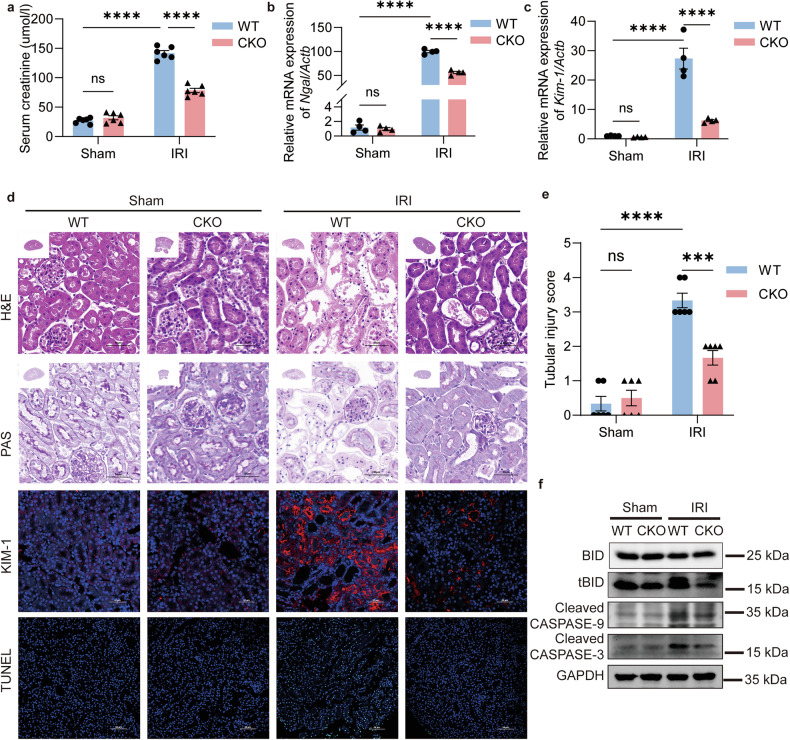
SENP3 deficiency attenuates renal ischemia-reperfusion injury. Mice underwent right nephrectomy followed by 30 min of left renal pedicle clamping and 24 h of reperfusion (IRI group) or sham surgery (Sham group). **a** Serum creatinine levels were lower in *Senp3* CKO mice than in WT controls after IRI. **b**, **c** RT-qPCR showed reduced mRNA expression of injury markers *Ngal* and *Kim-1* in kidneys from CKO mice compared with WT mice after IRI. **d** Representative images of H&E and PAS staining, KIM-1 immunofluorescence, and TUNEL apoptosis assay in kidney sections. *Senp3* CKO mice displayed less severe tubular injury. Scale bar, 50 μm. **e** Quantification of tubular injury scores. **f** Western blot analysis of apoptosis-related proteins in kidney lysates. Data: mean ± SEM (*n* = 6). ns, not significance. ***p < 0.001, ****p < 0.0001.

To examine the role of SENP3 in PTECs, we generated proximal tubule-specific *Senp3* knockout mice (*Senp3*^*flox/flox*^-*Ggt1*^*Cre*^, referred to as CKO) and control littermates (*Senp3*^*flox/flox*^ mice, WT). Successful deletion of SENP3 in PTECs of CKO mice was confirmed by PCR genotyping, Western blot analysis, and immunofluorescence staining (Fig. S3). No significant difference in renal injury was observed between sham-operated CKO and WT mice. Following IRI, CKO mice exhibited significantly attenuated renal injury compared with WT controls, as indicated by lower serum creatinine levels, reduced expression of neutrophil gelatinase-associated lipocalin (NGAL) and kidney injury molecule-1 (KIM-1) (Fig. 2a–c), and fewer TUNEL-positive apoptotic cells, and fewer TUNEL-positive apoptotic cells (Fig. 2d, e; Fig. S4a–b).

### SENP3-mediated DeSUMOylation activates the intrinsic apoptosis pathway

To elucidate the molecular mechanism underlying SENP3-dependeny apoptosis, we analyzed key components of apoptotic signaling pathways. In renal tissues following IRI, deficiency of SENP3 significantly attenuated the activation of BID and its truncated form (tBID), as well as the cleavage of CASPASE-9, cleaved CASPASE-3, but not that of CASPASE-8 (Fig. 2f; Fig. S4c–f, Fig. S8a–b).

**Fig. 3:**
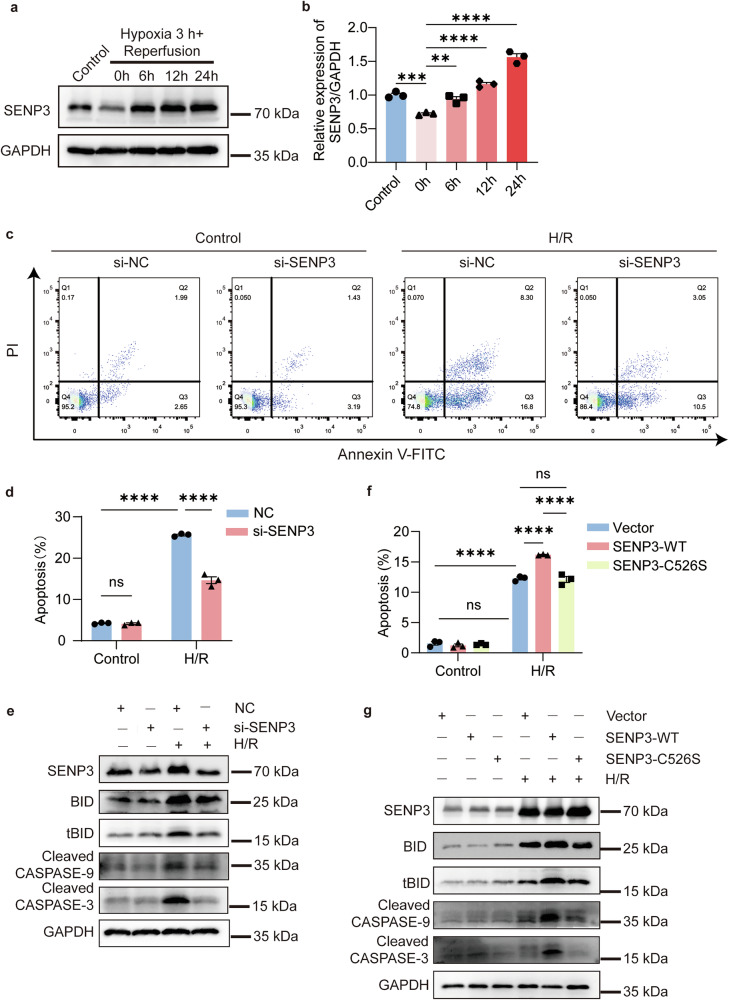
SENP3 deSUMOylase activity promotes H/R-induced apoptosis. TCMK1 cells were subjected to 3 h hypoxia followed by 24 h of reoxygenation (H/R). **a**–**b** SENP3 protein expression exhibited dynamic changes during reoxygenation, peaking at 24 h. **c**, **d** Apoptosis was assessed by flow cytometry with Annexin V-FITC/PI in cells transfected with SENP3-targeting siRNA (si-SENP3) or negative control (NC) siRNA after H/R. **e** Western blot analysis of apoptosis-related markers in si-SENP3 and NC cells under the same H/R conditions. **f** Overexpression of wild-type SENP3 (SENP3-WT), but not the catalytically inactive mutant SENP-C526S, exacerbated H/R induced cell apoptosis as measured by Annexin V-FITC/PI staining. **g** Corresponding western blot analysis of apoptosis-related proteins in cells overexpressing SENP3-WT or SENP3-C526S after H/R. Data: mean ± SEM (*n* = 3). ns, not significance. **p < 0.01, ***p < 0.001, ****p < 0.0001.

These findings were further validated in vitro. After 3 h of hypoxia, SENP3 transcription increased progressively with extended reoxygenation (Fig. S5), while its protein expression decreased transiently before showing a progressive increase during reoxygenation (Fig. 3a, b). Transfection of TCMK1 cells with SENP3 siRNA conferred significant cytoprotection against apoptosis (Fig. 3c, d; Fig. S6), consistent with the in vivo observations. Similarly, SENP3 knockdown suppressed the activation of apoptotic executors, with the exception of CASPASE-8 cleavage (Fig. 3e; Fig. S7 and S8b).

To establish the requirement for SENP3’s deSUMOylation activity, we generated constructs expressing wild-type SENP3 (SENP3-WT-HIS) and a catalytically inactive mutant (SENP3-C526S-HIS), which contains a cysteine-to-serine substitution at residue 526 (Fig. S9) [21]. Overexpression of SENP3-WT, but not the C526S mutant, exacerbated H/R-induced apoptosis (Fig. 3f, Fig. S10a) and promoted the generation of BID/tBID as well as the cleavage of CASPASE-9 and CASPASE-3 (Fig. 3g, Fig. S10b–e). These results demonstrate that SENP3 promotes renal tubular apoptosis through its deSUMOylating activity, primarily via activation of the intrinsic apoptosis pathway.

### Identification of SUMO2/3-Modified ASS1 as a Key SENP3 Substrate in Renal IRI

Building on the finding that SENP3-mediated deSUMOylation promotes apoptosis, we sought to identify critical SUMO2/3-conjugated protein substrates involved in IRI pathogenesis using a comprehensive proteomic approach. We first immunoprecipitated SUMO2/3 modified proteins from kidney lysates of IRI-AKI mice with or without SENP3 deficiency and identified 16 potential SUMO2/3 targets by quantitative mass spectrometry (Fig. S11). We compared the biological functions and renal tissue distribution of these candidates (Table S3) and evaluated their reported relevance to disease mechanisms. Arginosuccinate synthase 1 (ASS1), a rate-limiting enzyme in arginine biosynthesis within the urea cycle, is highly expressed in the kidney, particularly in proximal tubule cells. Previous studies have shown that ASS1 expression inhibits fibroblast proliferation [22], and ASS1 loss with consequent arginine auxotrophy has been observed in multiple tumor types; conversely, ASS1 overexpression suppresses tumor growth and promotes apoptosis [23]. Based on this evidence, we selected ASS1 for further investigation (Fig. S12).

**Fig. 4:**
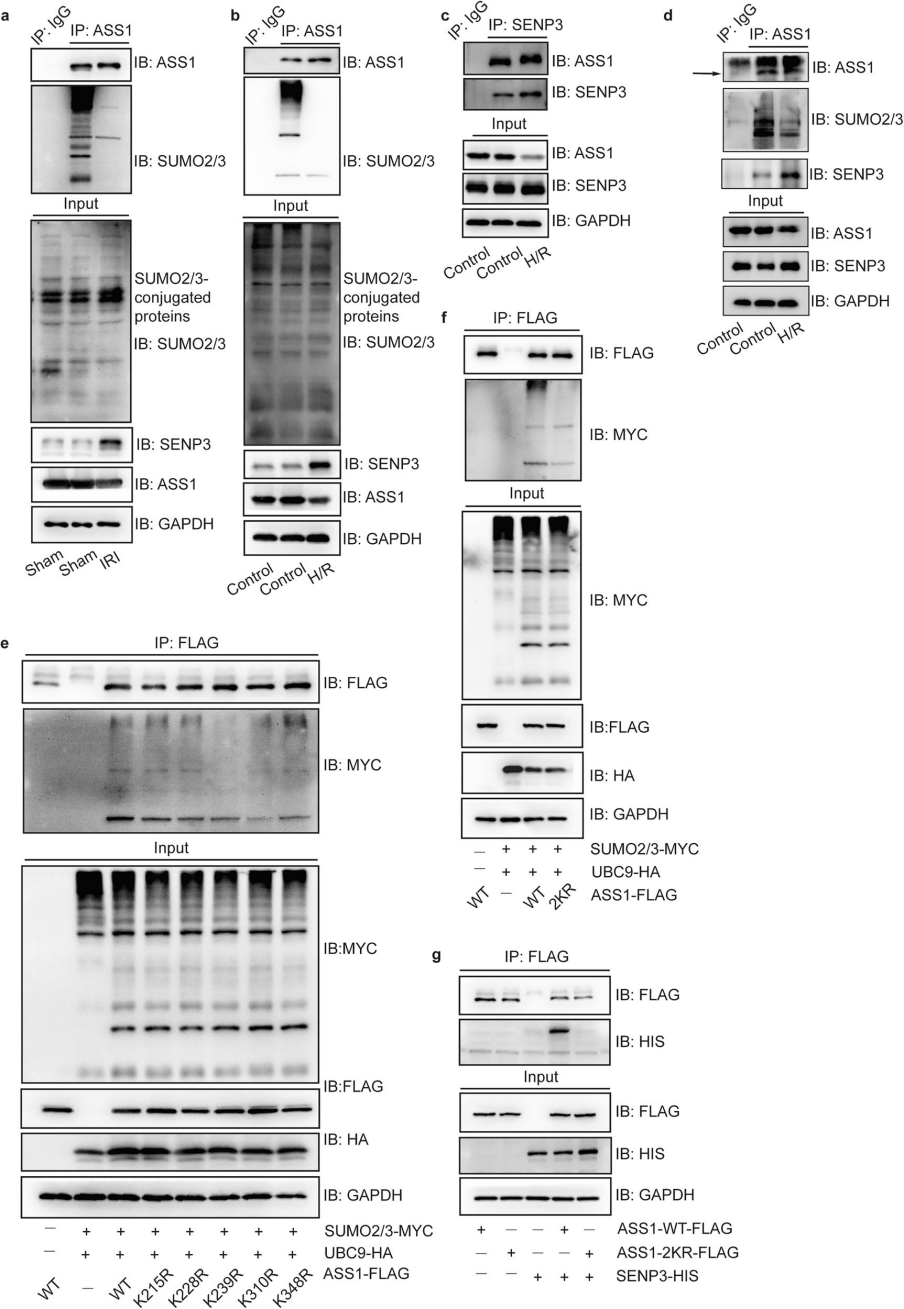
SENP3 mediates ASS1 deSUMOylation at K239 and K310 during H/R. **a**, **b** The interaction between SUMO2/3 and ASS1 was reduced in IRI kidney tissue and H/R-treated TCMK1 cells. **c**, **d** Endogenous binding between SENP3 and ASS1 in TCMK1 cells was enhanced following H/R. **e**, **f** SUMO2/3 conjugation occurred preferentially at lysine residues K239 and K310 of ASS1, as the double mutant ASS1-2KR(K239R/K310R) abrogated this modification. **g** The ASS1-2KR mutation impaired the interaction between SENP3 and ASS1. For co-immunoprecipitation assays in (**c**–**g**), HEK-293T cells were transfected for 48 h with expression vectors encoding ASS1, UBC9 (an essential E2-conjugating enzyme in the SUMOylation process), and SUMO2/3. All experiments were repeated in at least three biological replicates.

We confirmed that ASS-SUMO2/3 conjugation decreased in WT kidneys after IRI (Fig. 4a) and recapitulated this finding in TCMK1 cells (Fig. 4b). Similarly, ASS1 was found to interact with SENP3, while H/R treatment significantly reduced ASS1 SUMOylation and concurrently enhanced the ASS1-SENP3 interaction. (Fig. 4c, d). Since IRI and H/R appeared to reduce ASS1 protein levels, we assessed ASS1 transcription and translation during reoxygenation and observed an initial increase followed by a decline (Fig. S13).

To identify potential SUMOylation sites in mouse ASS1 (NP_031520.1, P16460), we used three predicting tools: SUMOplot, JASSA and GPS-SUMO (Fig. S14a–c). Given the high sequence homology between human and mouse and previously reported SUMOylation sites in human *ASS1* [24], we aligned their amino acid sequences and cross-referenced known sites with our predictions (Fig. S14d). We selected five candidate lysine residues− K215, K228, K239, K310, and K348−for validation, mutating each to arginine (R) to assess its role in SUMOylation. Co-transfection experiments in HEK-293T cells confirmed the interaction among exogenous ASS1, SUMO2/3 and SENP3 (Fig. S15). Mutations at K239 and K310 markedly reduced SUMO2/3 modification levels, whereas mutations at K215, K228 and K348 had minimal effects (Fig. 4e). We then generated a FLAG-tagged ASS1 double mutant (K239R/K310R, termed 2KR) and co-expressed it with HA-UBC9, His-SENP3 or MYC-SUMO2/3 in HEK-293T cells. The 2KR mutation substantially diminished SUMO2/3 modification of ASS1 (Fig. 4f) and disrupted its interaction with SENP3 (Fig. 4g). In summary, our data demonstrates that ASS1 is modified by SUMO2/3 primarily at K239 and K310, and this modification is reversed by SENP3-mediated deSUMOylation.

### SENP3-dependent DeSUMOylation is required for ASS1 nuclear accumulation

**Fig. 5:**
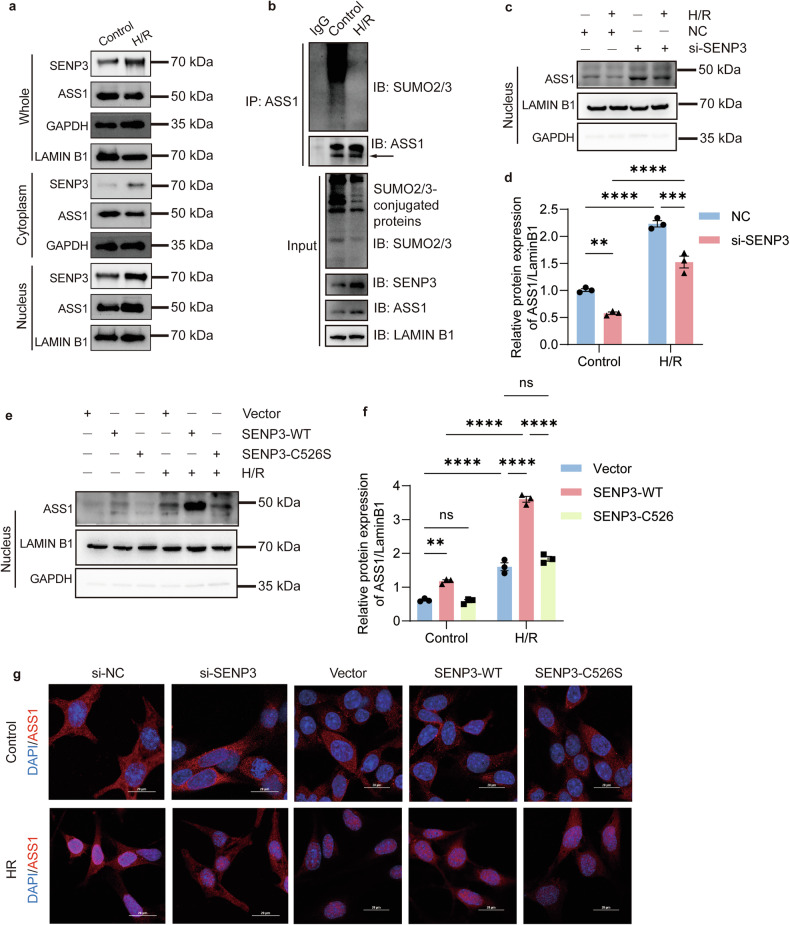
Nuclear accumulation of deSUMOylated ASS1 depends on SENP3 activity. **a**, **b** Following H/R treatment, TCMK1 cells showed increased nuclear accumulation of ASS1, which was predominantly in a deSUMOylation form, as demonstrated by co-immunoprecipitation of nuclear fractions. **c**, **d** Nuclear ASS1 levels in TCMK1 cells transfected with control (NC) or SENP3-targeting siRNA after H/R. **e**, **f** Nuclear ASS1 expression in TCMK1 cells transduced with lentivirus encoding wild-type SENP3 (SENP3-WT) or catalytically inactive SENP3-C526S after H/R. The SENP3-C526S mutant failed to promote ASS1 nuclear accumulation. **g** Immunofluorescence staining further confirmed that the nuclear translocation of ASS1 induced by H/R was dependent on SENP3 activity. Scale bar, 20 μm. Data: mean ± SEM *(n* = *3)*. ns, not significance. **p < 0.01, ***p < 0.001, ****p < 0.0001.

Under basal conditions, ASS1 localizes to both the cytosol and nucleus, whereas SENP3 and SUMO2/3 are predominantly nuclear. Following H/R treatment, ASS1 accumulation in the nucleus increased, accompanied by a decrease in its cytosolic level, while SENP3 expression was elevated in both compartments (Fig. 5a**;** Fig. S16a–d). Notably, nuclear ASS1 was primarily in a deSUMOylated state (Fig. 5b). To determine whether SENP3-mediated deSUMOylation regulates ASS1 nuclear translocation, we performed subcellular fractionation assays. Knockdown of SENP3 via siRNA in TCMK1 cells reduced nuclear ASS1 levels and partially suppressed H/R-induced nuclear accumulation of ASS1 (Fig. 5c, d, Fig. S16e-f). Conversely, SENP3 overexpression further enhanced nuclear ASS1 accumulation. However, overexpression of the catalytically inactive mutant SENP3-C526S failed to promote ASS1 nuclear translocation, showing effects comparable to basal SENP3 levels (Fig. 5e, f, Fig. S16g–h). Immunofluorescence staining further confirmed that the nuclear translocation of ASS1 induced by H/R was dependent on SENP3 activity (Fig. 5g). Consistent with these findings, ASS1 nuclear accumulation was also observed in renal PTECs following IRI (Fig. S17).

**Fig. 6:**
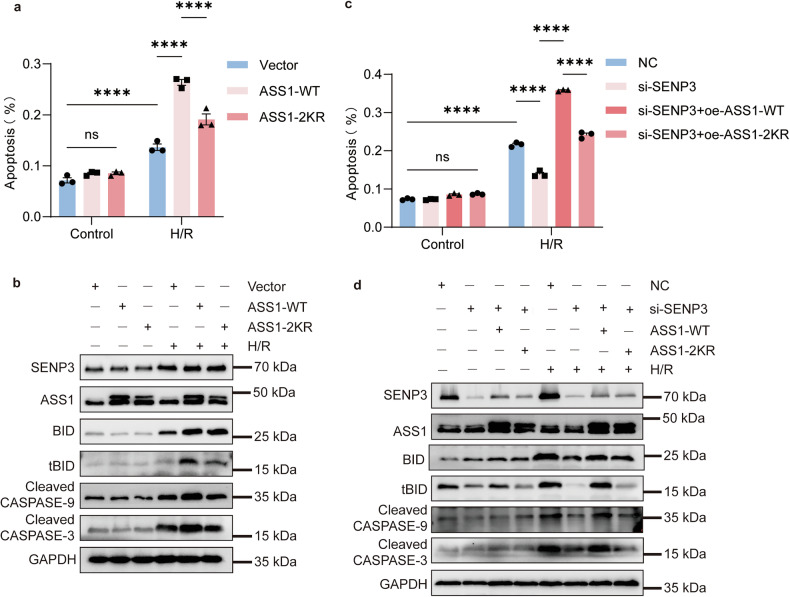
ASS1 deSUMOylation is required for the pro-apoptotic function of SENP3. **a**, **b** TCMK1 cells were transfected with wild-type ASS1 (ASS-WT) or the SUMOylation-deficient mutant (ASS1-2KR) for 24 h and then subjected to H/R. Flow cytometry analysis (Annexin V-FITC/PI) and Western blotting of apoptosis-related proteins showed that ASS1-WT promoted apoptosis more strongly than ASS1-2KR. **c**, **d** TCMK1 cells were co-transfected with SENP3 siRNA and either ASS1-WT or ASS1-2KR for 24 h, followed by H/R. Apoptosis analysis and Western blot of apoptotic markers revealed that ASS1-WT, but not ASS1-2KR, significantly enhanced apoptosis even under SENP3-deficient conditions. Data: mean ± SEM (*n* = 3). ns, not significance. ****p < 0.0001.

As predicted, overexpression of wild-type ASS1 enhanced H/R-induced apoptosis, whereas the pro-apoptotic effect of the SUMOylation-deficient ASS1-2KR mutant was attenuated compared to the wild-type protein (Fig. 6a, b, Fig. S18). To further examine the functional role of deSUMOylated ASS1 in H/R-induced apoptosis, we conducted rescue experiments. Under SENP3 knockdown conditions, restoration of wild-type ASS1 promoted apoptosis, while the SUMOylation site-mutant failed to exert such pro-apoptosis effects (Fig. 6c, d, Fig. S19). Collectively, these results demonstrate that H/R-induced nuclear accumulation of ASS1 in TCMK1 cells, promoting apoptosis, is dependent on SENP3-mediated deSUMOylation.

### ASS1 DeSUMOylation induced apoptosis by enhancing p53 transcriptional activity

**Fig. 7:**
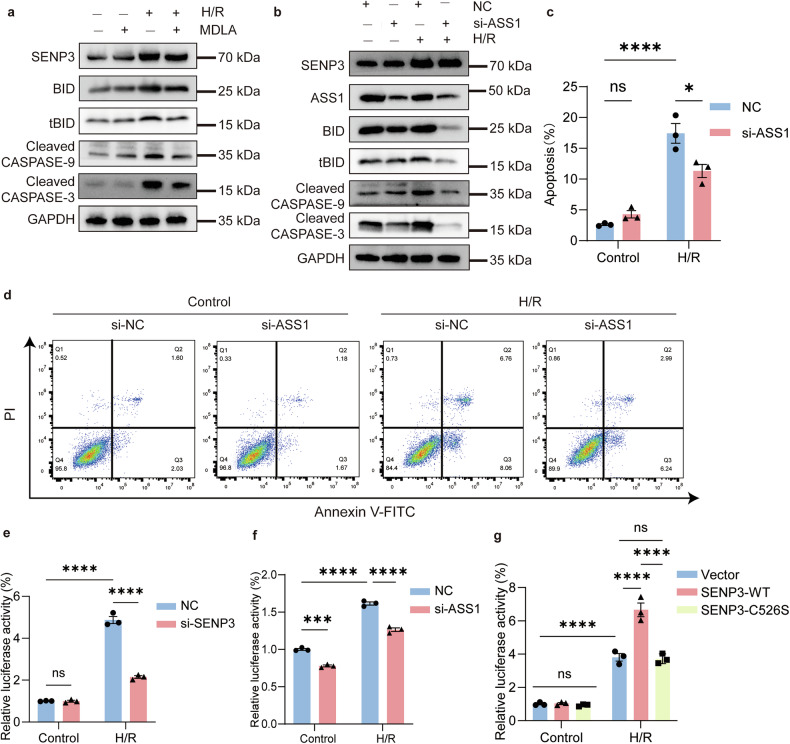
ASS1 inhibition mitigates H/R-induced apoptosis. **a** TCMK1 cells were pretreated with the ASS1 Inhibitor MDLA (20 mM, 24 h) prior to H/R. MDLA reduced the expression of apoptosis-related proteins following H/R. **b**–**d** ASS1 knockdown in TCMK1 cells attenuated H/R-induced apoptosis, as assessed by Annexin-V-FITC/PI flow cytometry, and decreased the expression of apoptosis-related proteins. **e** Knockdown of SENP3 attenuated *Trp53* transcriptional activity in TCMK1 cells under H/R conditions. **f** ASS1 knockdown suppressed H/R-induced *Trp53* transcriptional activation. **g** The catalytically inactive SENP3 mutant (C526S) failed to enhance *Trp53* transcriptional activity following H/R. Data: mean ± SEM (*n* = 3). ns, not significance. *p < 0.05, ***p < 0.001, ****p < 0.0001.

Based on our findings, ASS1 accumulates in the nucleus following IRI or H/R in a SENP3-dependent deSUMOylation manner. Notably, pharmacological inhibition of ASS1 using MDLA attenuated H/R-induced apoptosis, even in the absence of SENP3 deficiency (Fig. 7a**;** Fig. S20). To further validate these observations, we knockdown ASS1 in TCMK1 cells using siRNA. As expected, ASS1 silencing markedly alleviated H/R-induced apoptosis (Fig. 7b–d). Similarly, ASS1-deficient TCMK1 cells exhibited reduced expression of pro-apoptotic proteins (Fig. S21).

While these results establish a clear association between nuclear deSUMOylated ASS1 and apoptosis, the underling molecular mechanism required further investigation. Previous studies have demonstrated that severe hypoxia triggers p53 accumulation, which regulates downstream targets to promote mitochondria-dependent apoptosis; notably, BID expression is transcriptionally regulated by p53 [25]. We therefore hypothesized that nuclear deSUMOylated ASS1 enhanced apoptosis by promoting *Trp53* transactivation. H/R treatment increased the co-localization of p53 and ASS1 (Fig. S22a) and enhanced *Trp53* transcriptional activity, effects that were attenuated by either SENP3 or ASS1 knockdown (Fig. 7e, f). Conversely, SENP3 overexpression further amplified *Trp53* transcription following H/R treatment, whereas the catalytically inactive SENP3-C526S mutant had an effect comparable to that of SENP3-WT (Fig. 7g). In vivo, SENP3 deficiency reduced p53 expression in renal tissue following IRI (Fig. S22b).

**Fig. 8:**
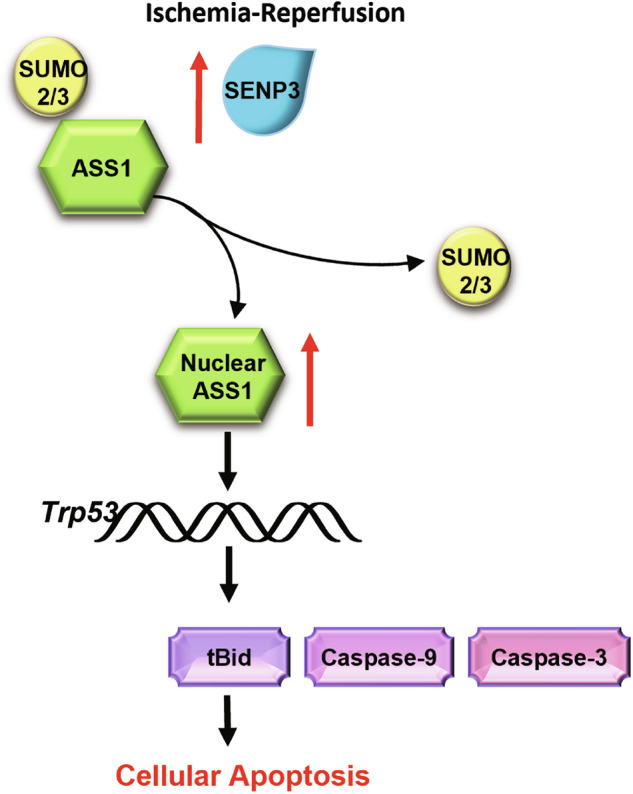
Proposed model for the role of ASS1 deSUMOylation in renal IRI. Renal ischemia/reperfusion promotes SENP3-mediated deSUMOylation of ASS1, facilitating its nuclear accumulation and enhances *Trp53* transcriptional activity, ultimately resulting in tubular epithelial cell apoptosis.

Complementary mRNA sequencing analysis of H/R-treated TCMK1 cells revealed significant upregulation of *Trp53* and its downstream target *Bid* (Fig. S22c–d). These findings were further confirmed by RT-qPCR, which demonstrated that *Bid* expression was regulated in a SENP3 deSUMOylation-dependent manner consistent with *Trp53* transcriptional activation (Fig. S23). In summary, our data demonstrate that SENP3-mediated deSUMOylation of ASS1 promotes its nuclear accumulation, which in turn enhanced *Trp53* transcriptional activity and ultimately induces apoptosis following IRI (Fig. 8).

## Discussion

Protein SUMOylation plays critical roles in pathophysiology of various diseases and represents a promising therapeutic target. In this study, we identified ASS1 as a novel SUMO-modified protein involved in renal IRI. Our findings demonstrate that SENP3-mediated deSUMOylation of SUMO2/3-conjugated ASS1 promotes apoptosis in PTECs following ischemic or hypoxic stress.

The functional consequences of SUMOylation in AKI appear to be context-dependent, varying with etiology, specific SUMO isoforms, regulatory SENP proteases, and substrate proteins. Previous studies have reported dynamic changes in protein SUMOylation during ischemic AKI, showing that SUMO1- and SUMO2/3-modified proteins increasing modestly after 30 min of ischemia but dramatically after 8 h of reperfusion, before declining during the 24-48-hour perfusion period [17]. In both folic acid- and IRI induced-AKI models, increased SUMO1 conjugation to Sirt3 was observed, and SENP1-mediated deSUMOylation of Sirt3 alleviated kidney injury [18]. Interesting, while global SUMOylation inhibition sensitized renal cells to apoptosis in cisplatin-induced nephrotoxic [17], and SENP1 deficiency exacerbated cisplatin-induced renal damage [26], our study reveals a cytoprotective role for SUMO2/3-modified ASS1 in SENP3-deficient IRI-AKI model, underscoring the complex and substrate-specific nature of SUMOylation in renal pathophysiology.

Emerging evidence implicates SUMOylation, particular SUMO2/3 conjugation, in cellular responses to ischemic and hypoxic stress [27, 28]. Although SUMO2 and SUMO3 share near-identical sequence and are often referred to collectively as SUMO2/3, SUMO2 is a more abundant isoform than SUMO3 in mammals, and can compensate for most SUMO1 function [29]. These observations highlight the importance of identifying specific SUMO2/3 target proteins in renal IRI. Our findings suggesting that enhancing ASS1 SUMOylation may represent a novel therapeutic strategy for IRI-induced AKI.

Under basal conditions, most SUMO targets exhibit low modification levels but undergo rapid SUMOylation cycle in where even minimal modifications can exert significant effects. Nuclear SUMOylation generally suppresses transcription by modifying transcription factors and co-regulators [30]. However, exceptions exist-for instance, oxidative stress-induces SUMOylation of TP53INP1 is required for p53 activation and subsequent apoptosis [31]. In cerebral IRI, blocking ANXA1 deSUMOylation reduces apoptosis by inhibiting p53 transcriptional activity [32, 33]. Our study adds further nuance to this picture by showing that nuclear accumulation of deSUMOylated ASS1 following H/R enhances p53 transcriptional activity.

Several limitations of our study warrant consideration. First, although SENP3 and SENP5 were significantly upregulated following IRI and H/R, we selected SENP3 for further investigation and identified ASS1 as one of its substrates involved in the IRI process. We acknowledge that other SENP isoforms may also regulate ASS1. Second, although we identified 16 potential SUMO2/3-modified proteins in IRI-induced apoptosis, we focus exclusively on ASS1 based on bioinformatic analysis; other candidates may also contribute to this process. Third, although we established SENP3-dependent nuclear accumulation of deSUMOylated ASS1 post-H/R, the precise molecular mechanisms require further investigation.

In conclusion, our study underscores the critical importance of SUMOylation homeostasis in renal protection and identifies SUMO2/3-modified ASS1 as a key regulator in IRI-induced AKI. We propose a novel apoptotic pathway in PTECs in which ischemia/reperfusion triggers SENP3-mediated ASS1 deSUMOylation, leading to nuclear accumulation and enhanced p53 transcriptional activation. These findings provide new insights into the molecular mechanisms of renal IRI and suggest potential therapeutic targets for AKI intervention.

## Supplementary information

supplemental material

## Data Availability

The mass spectrometry data have been deposited in the Integrated Proteome Resources (iProX: IPX0013721000), and the RNA-sequencing date are available in the National Genomics Data Center (NGDC: PRJCA047501/CRA050555) of China.
