# Supplementary material for: SENP3 promotes renal tubular epithelial cell apoptosis after ischemia-reperfusion injury via ASS1 deSUMOylation
Source: Cell Death Dis. 2025 Dec 5;17(1):76. doi: 10.1038/s41419-025-08308-2 (PMC12827350; doi:10.1038/s41419-025-08308-2)

Supplemental materials

[**Figure S1 Transcript levels of SENP family members in the kidney following IRI-AKI.** 3](#_Toc212226496)

[**Figure S2 Transcript levels of SENP family members in TCMK1 cells following H/R.** 3](#_Toc212226497)

[**Figure S3 Validation of successful SENP3 knockout in renal tubular cells.** 4](#_Toc212226498)

[**Figure S4 SENP3 deficiency attenuates renal IRI.** 5](#_Toc212226499)

[**Figure S5 SENP3 transcriptional increased after H/R.** 6](#_Toc212226500)

[**Figure S6 Efficient knockdown of SENP3 in TCMK1 cells.** 6](#_Toc212226501)

[**Figure S7 SENP3 knockdown suppressed H/R-induced apoptosis in TCMK1 cells.** 7](#_Toc212226502)

[**Figure S8 SENP3 deficiency does not reduce cleaved CASPASE-8 expression following renal IRI or H/R treatment.** 8](#_Toc212226503)

[**Figure S9 Sequence alignment of human and mouse SENP3 catalytic domains.** 9](#_Toc212226504)

[**Figure S10 SENP3 deSUMOylase activity promotes H/R-induced apoptosis.** 11](#_Toc212226505)

[**Figure S11 Identification of SUMO2/3-modified proteins in renal IRI.** 11](#_Toc212226506)

[**Figure S12 Secondary Mass Spectrometry of ASS1 (Protein ID: P16460).** 12](#_Toc212226507)

[**Figure S13 Dynamic changes in ASS1 expression following H/R.** 13](#_Toc212226508)

[**Figure S14 Computational prediction and cross-species conservation of ASS1 SUMOylation sites** 15](#_Toc212226509)

[**Figure S15 Exogenous interactions between ASS1, SUMO2/3, and SENP3.** 16](#_Toc212226510)

[**Figure S16 Subcellular localization of ASS1 and SENP3 in TCMK1 cells under H/R conditions.** 17](#_Toc212226511)

[**Figure S17 The ASS1 accumulated in nuclear after IRI in PTECs.** 17](#_Toc212226512)

[**Figure S18 SUMOylation-deficient ASS1 attenuates H/R-induced apoptosis.** 18](#_Toc212226513)

[**Figure S19 ASS1 deSUMOylation is required for the pro-apoptotic function of SENP3 under H/R conditions.** 20](#_Toc212226514)

[**Figure S20 ASS1 Inhibitor MDLA attenuates H/R-induced apoptosis in TCMK1 cells.** 21](#_Toc212226515)

[**Figure S21 ASS1 deficiency attenuates H/R-induced apoptosis in TCMK1 cells.** 22](#_Toc212226516)

[**Figure S22 Hypoxia/reoxygenation (H/R) induces Trp53-mediated transcriptional activation of Bid.** 24](#_Toc212226517)

[**Figure S23 The transcription of Bid.** 24](#_Toc212226518)

[**Table S1 The details of primary antibodies.** 25](#_Toc212226519)

[**Table S2 The details of primers of RT-qPCR.** 25](#_Toc212226520)

[**Table S3 The characteristics of candidate proteins.** 26](#_Toc212226521)

[**The original figures of Western Blot.** 27](#_Toc212226522)


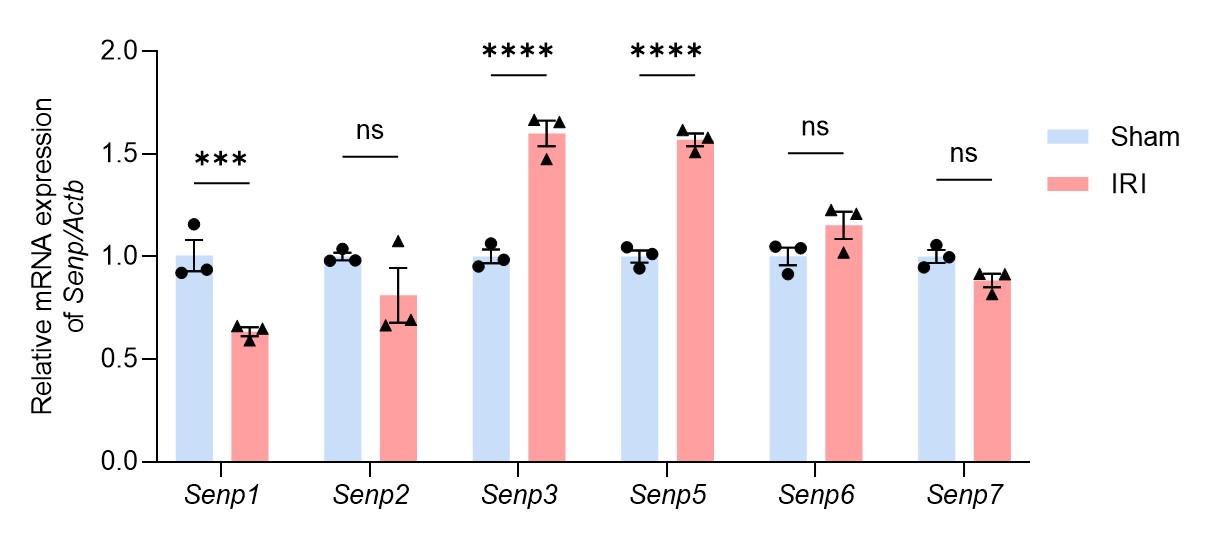


# **Figure S1 Transcript levels of SENP family members in the kidney following IRI-AKI.**

Mice underwent right nephrectomy followed by 30 min of left renal pedicle clamping and 24 h of reperfusion (IRI group) or sham surgery (Sham group). Renal tissues were harvested and analyzed by qPCR to evaluate SENP expression. Data: mean ± SEM (*n=3*); ***p < 0.001. ****p < 0.0001.


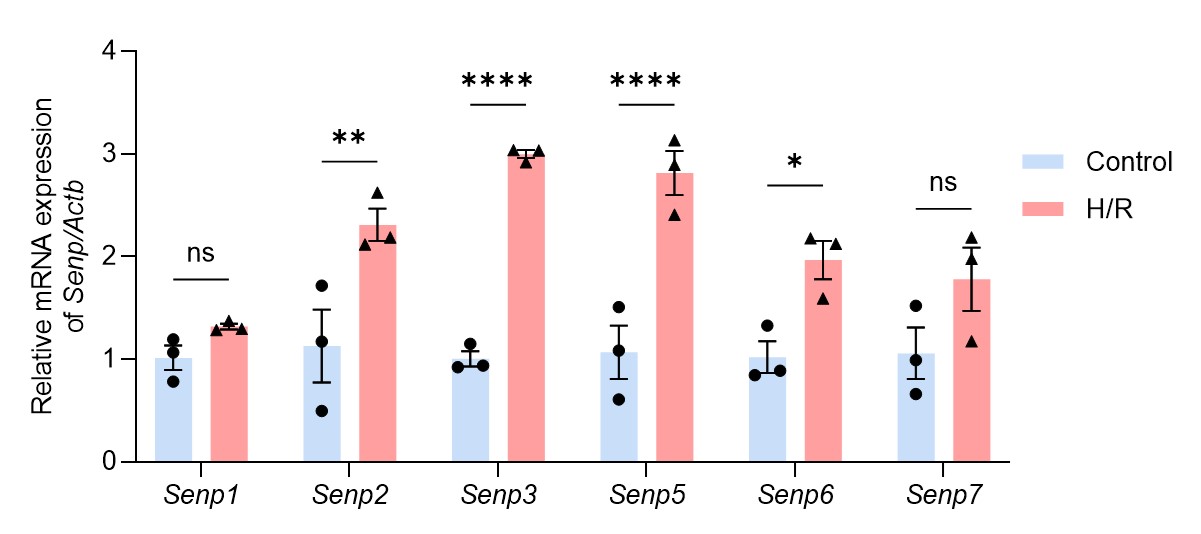


# **Figure S2 Transcript levels of SENP family members in TCMK1 cells following H/R.**

TCMK1 cells were subjected to 3 h of hypoxia followed by 24 h of reoxygenation. Total RNA was extracted from whole-cell lysates and analyzed by qPCR to determine SENP expression levels.. Data: mean ± SEM (*n=3*); *p < 0.05. **p < 0.01. ****p < 0.0001.


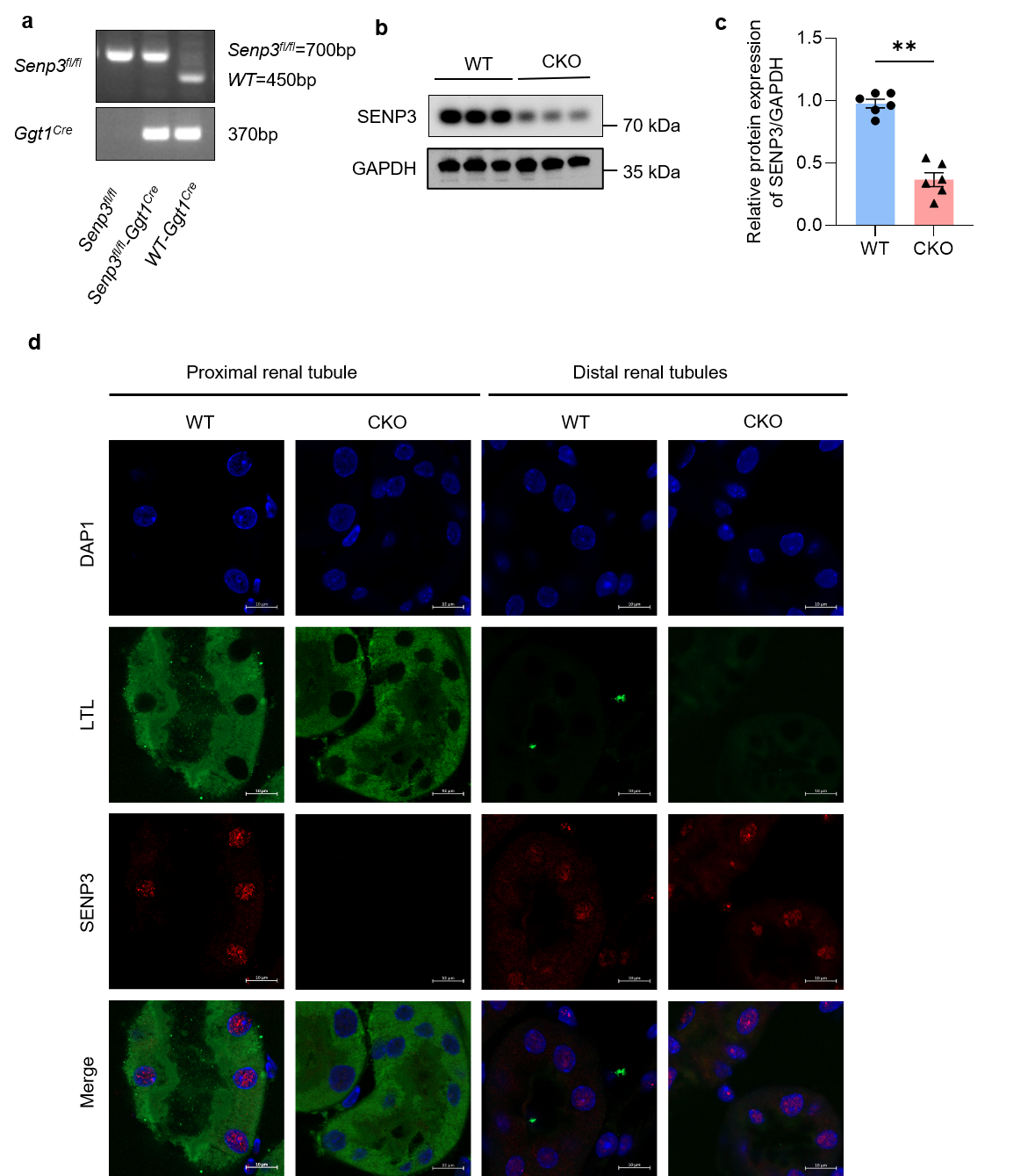


**Figure S3 Validation of successful SENP3 knockout in renal tubular cells.**

(a) PCR genotyping of *Senp3* and *Ggt1*using genomic DNA extracted from mouse toe tissue. (b-c) Western blot analysis showed significantly reduced SENP3 protein levels in renal tissues from Senp3 conditional knockout (CKO) mice compared with WT littermates. Data: mean ± SEM (*n=6*); **p < 0.01. (d) Immunofluorescence staining confirmed cell specific deletion of SENP3 in renal tubular cells. Scale bar: 10 µm.


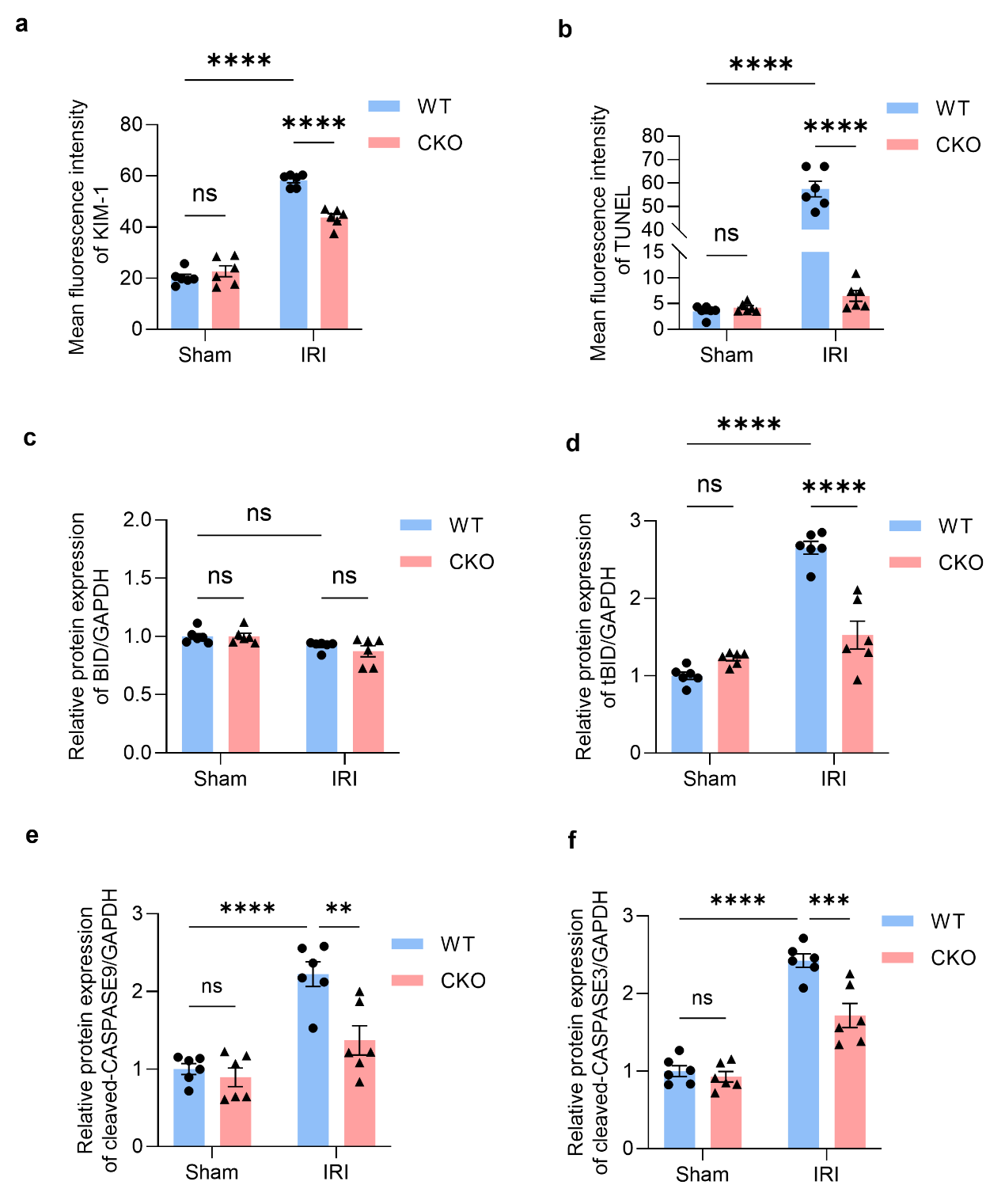


**Figure S4 SENP3 deficiency attenuates renal IRI.**

(a-b) RT-qPCR analysis of kidney tissues from IRI-AKI mice showed that SENP3 deficiency reduced the mRNA expression of injury markers *Ngal* and *Kim-1* following IRI. (c-f) Quantification analysis of apoptosis-related proteins (BID, tBID and cleaved CASPASE-9/3) in kidney lysates. Data: mean ±SEM (*n*=6). ns, not significance. *p <0.05, **p <0.01, ***p <0.001, ****p <0.0001.


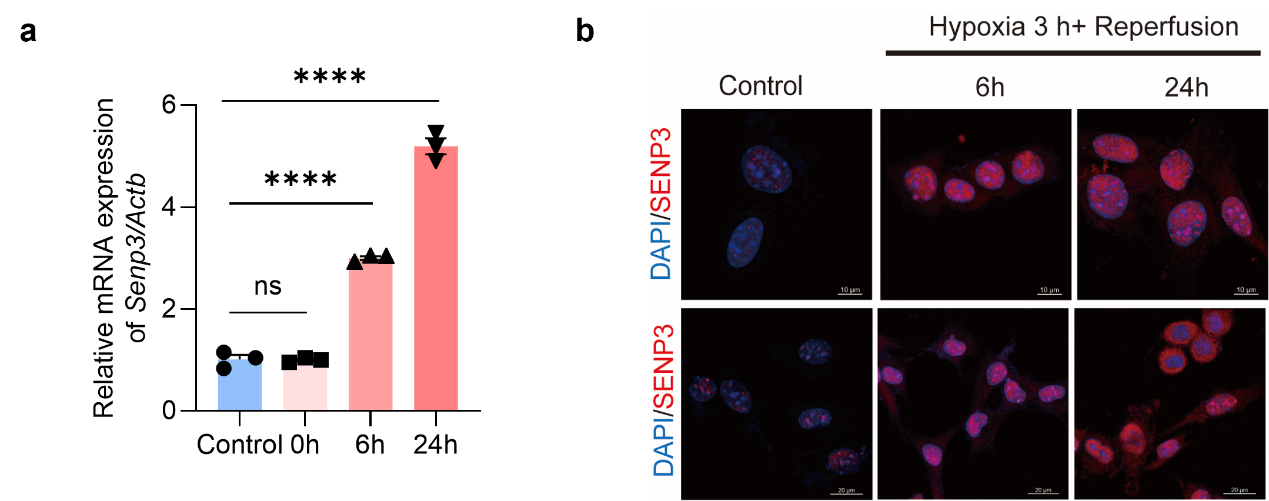


**Figure S5 SENP3 transcriptional increased after H/R.**

(a) The transcriptional level of *Senp3* in TCMK1 cells increased progressively with prolonged reoxygenation time. Data: mean ±SEM (*n*=3). ns, not significance. ****p <0.0001. (b) Immunofluorescence staining confirmed increased SENP3 (red) expression in TCMK1 cells after 6 hours and 24 hours reoxygenation. Scale bar, 10 μm (upper panels) and 20 μm (lower panels).


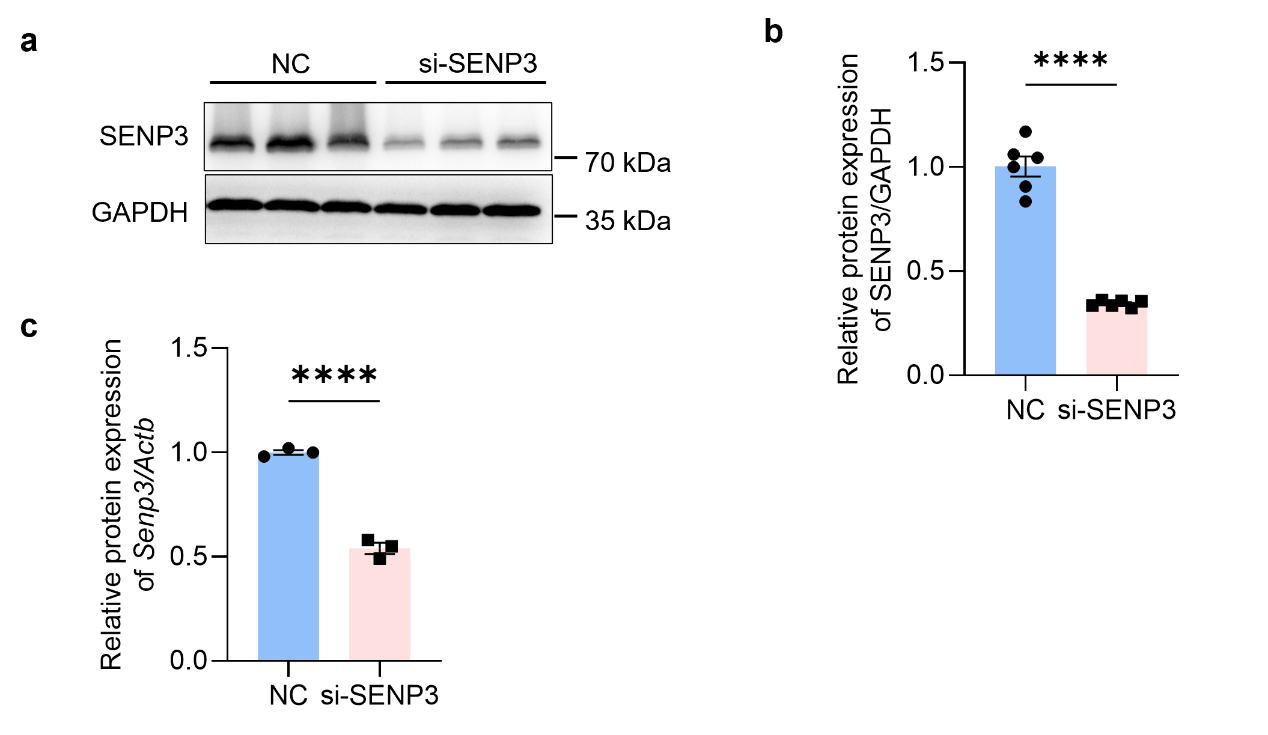


**Figure S6 Efficient knockdown of SENP3 in TCMK1 cells.**

(a-b) Western blot analysis and quantification showed a significant reduction in SENP3 protein levels in TCMK1 cells transfected with SENP3-targeting siRNA (si-SENP3) compared to negative control siRNA (NC). Data represent mean ± SEM (*n=6*). ****p < 0.0001. (c) RT-qPCR analysis confirmed effective transcriptional silencing of *Senp3* in siRNA-transfected cells. Data represent mean ± SEM (n=3). ****p < 0.0001.


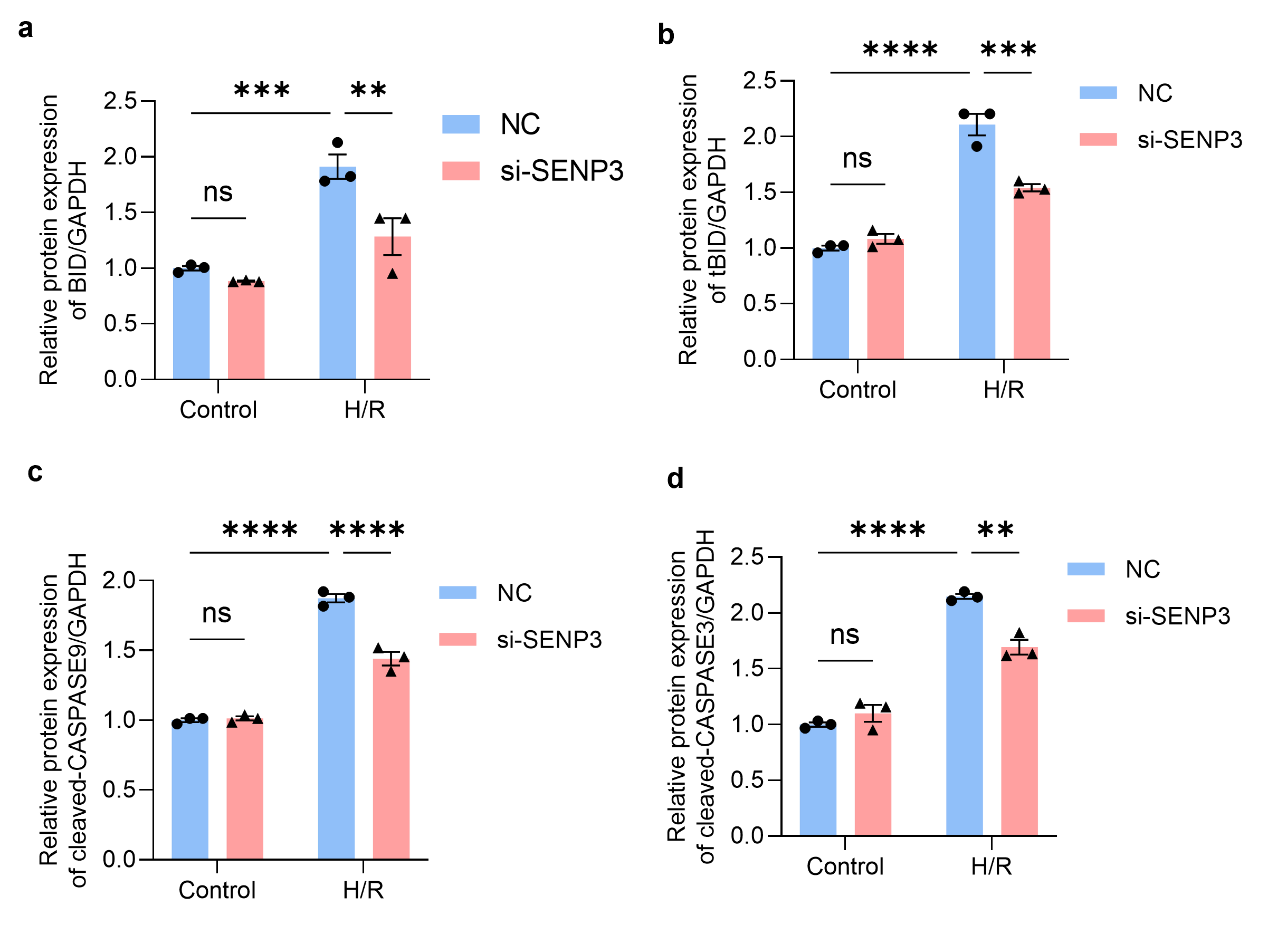


**Figure S7 SENP3 knockdown suppressed H/R-induced apoptosis in TCMK1 cells.**

(a-d) Quantitative analysis of apoptosis-related proteins (BID, tBID, and cleaved CASPASE-9/3) in TCMK1 cells following H/R treatment, with or without SENP3 knockdown. Data: mean ±SEM (n=3). ns, not significance. **p <0.01, ***p <0.001, ****p <0.0001.


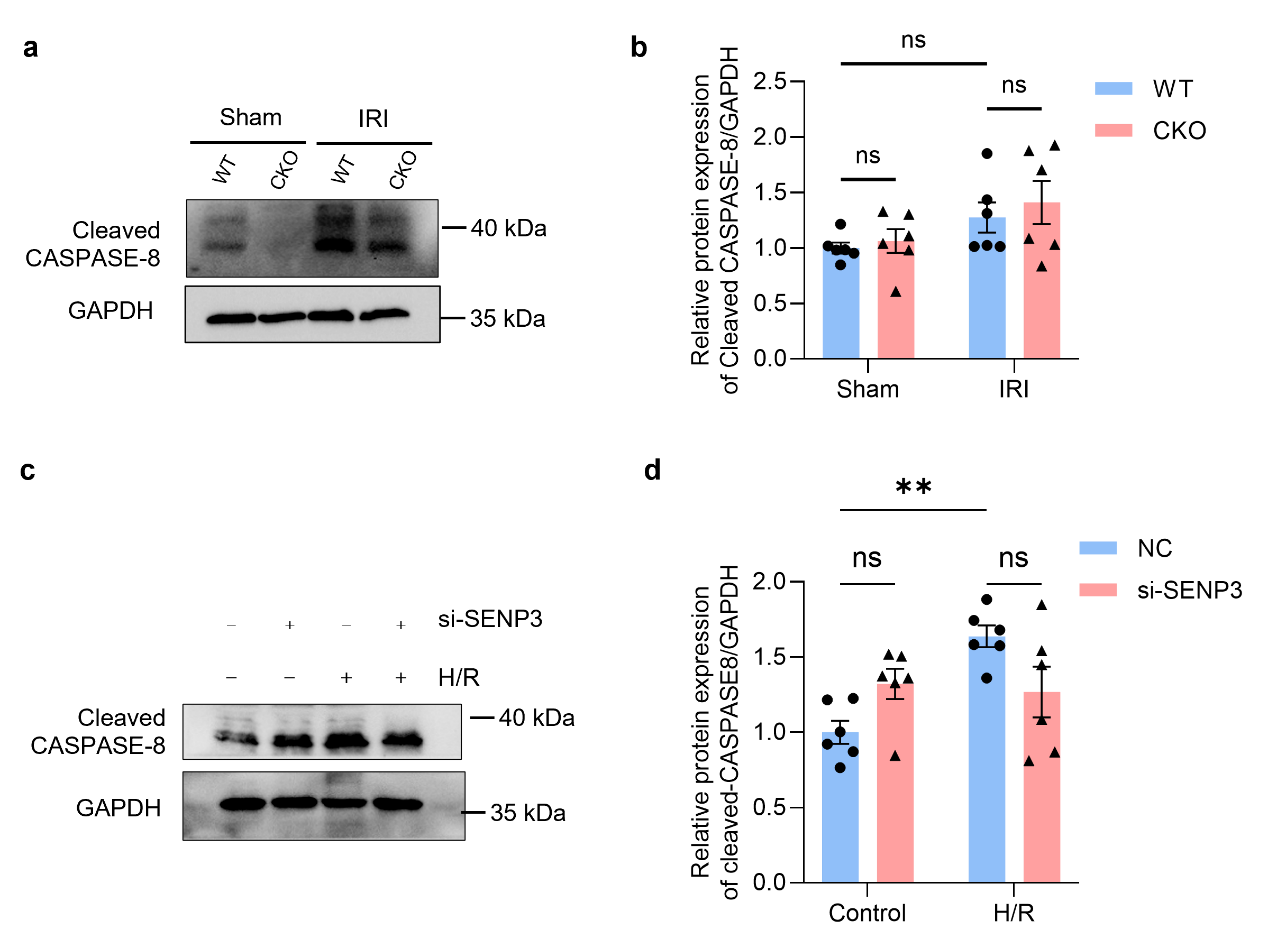


# **Figure S8 SENP3 deficiency does not reduce cleaved CASPASE-8 expression following renal IRI or H/R treatment.**

(a-b) Cleaved CASPASE -8 levels in kidney tissues of wild-type (WT) and *Senp3* conditional knockout (CKO) mice after ischemia-reperfusion injury (IRI). (c-d) Cleaved CASPASE-8 expression in TCMK1 cells subjected to H/R with or without SENP3 knockdown. Data represent mean ± SEM (*n=6*). ns, not significant. **p <0.01.


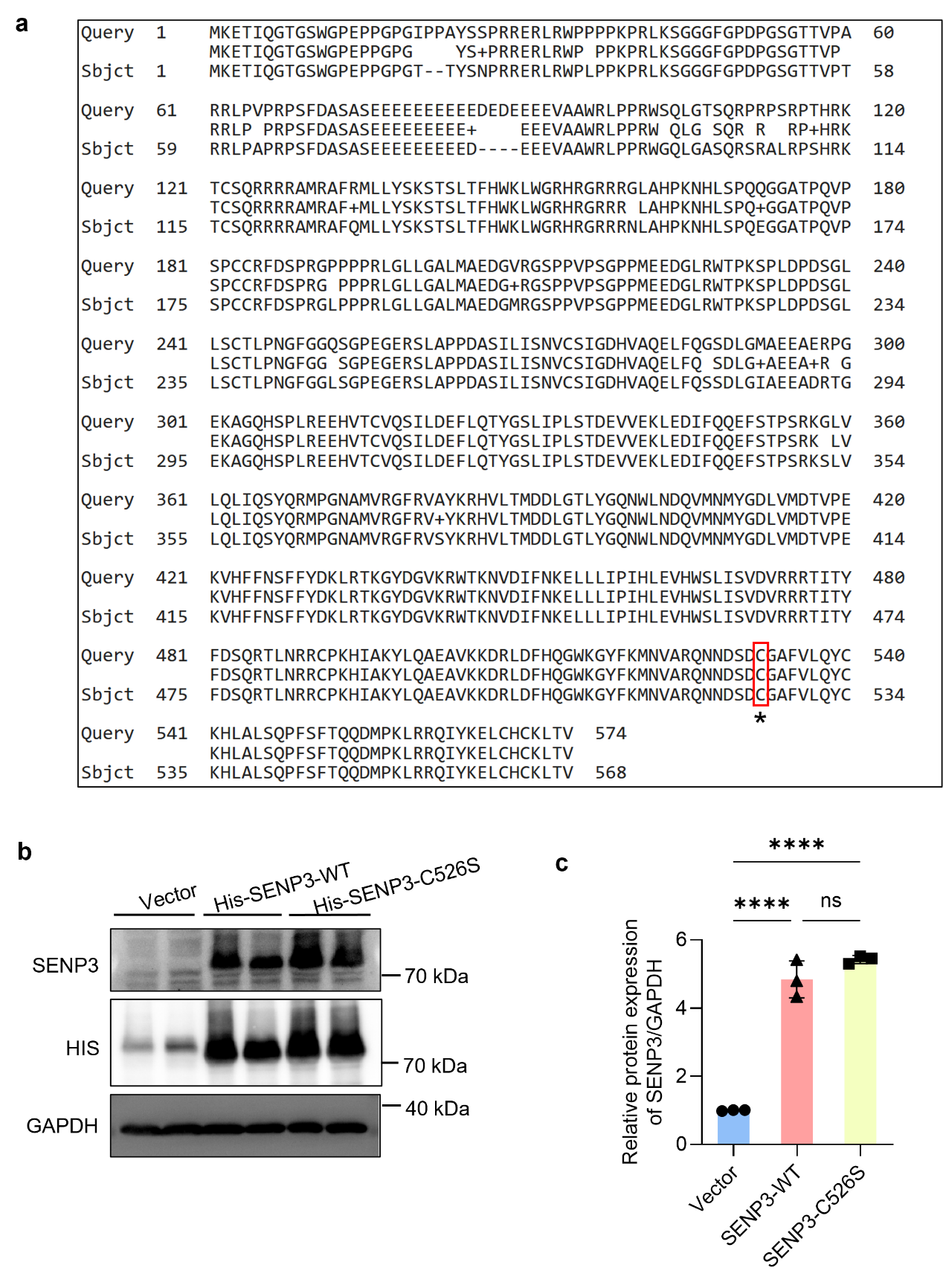


# **Figure S9 Sequence alignment of human and mouse SENP3 catalytic domains.**

(a) BLAST alignment shows conserved cysteine residues within the catalytic domains of human (top) and mouse (bottom) SENP3. The essential catalytic cysteine is located at position C532 in human SENP3 and C526 in mouse SENP3 (highlighted in red boxes). (b-c) Western blot analysis and quantification confirm comparable expression levels of His-tagged wild-type SENP3 (SENP3-WT) and catalytically inactive mutant (SENP3-C526S) in TCMK1 cells relative to empty vector control. Data represent mean ± SEM (*n=3*). ns, not significant; ****p < 0.0001.


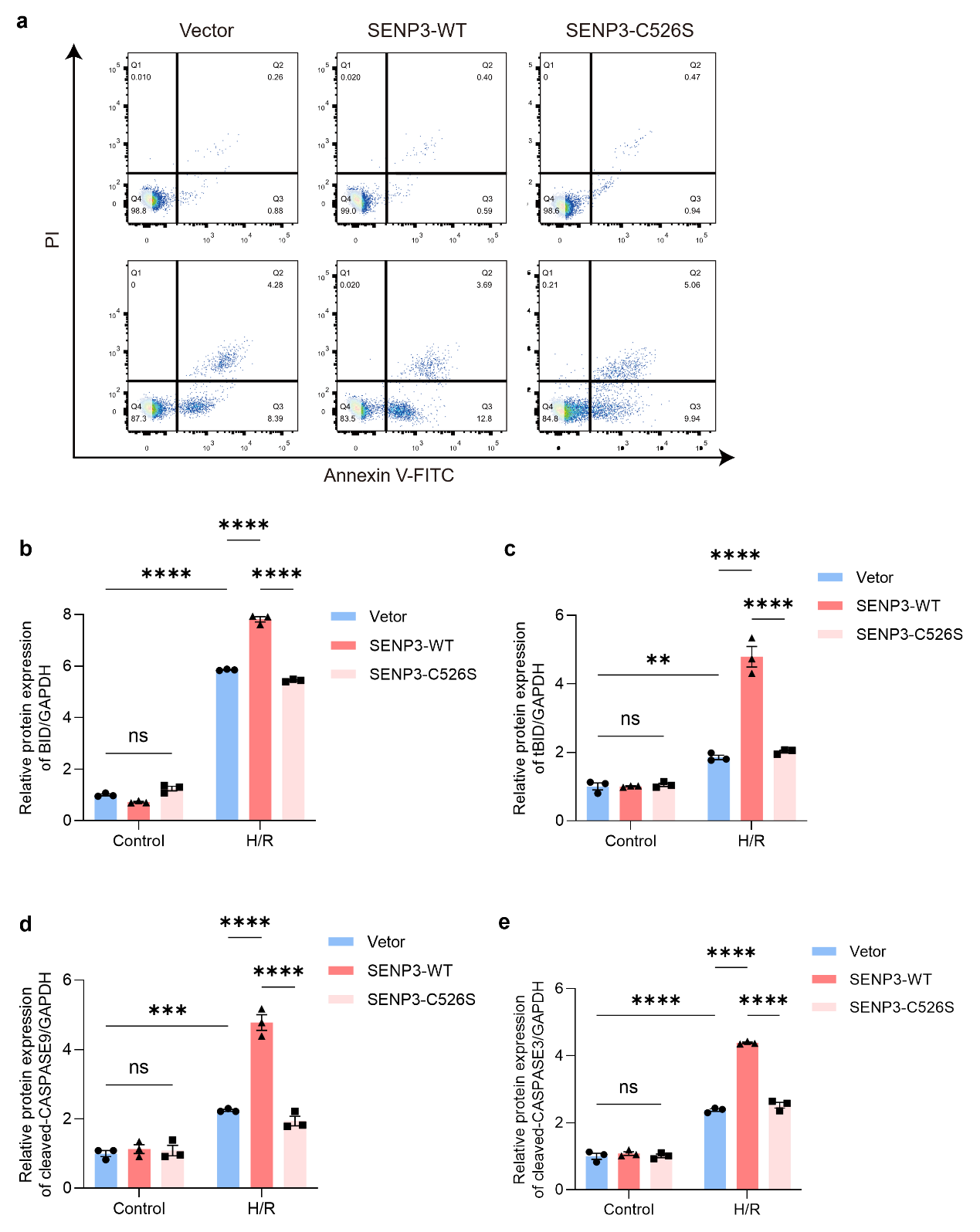


# **Figure S10 SENP3 deSUMOylase activity promotes H/R-induced apoptosis.**

(a) Overexpression of wild‑type SENP3 (SENP3‑WT), but not the catalytically inactive mutant (SENP3‑C526S), increased apoptosis as measured by Annexin V‑FITC/PI staining. (b-e) Expression levels of apoptosis‑related proteins were elevated in cells overexpressing SENP3‑WT, whereas the C526S mutation abrogated this effect. Data: mean ±SEM (*n=3*)*.* ns, not significance. **p <0.01, ***p <0.001, ****p <0.0001.


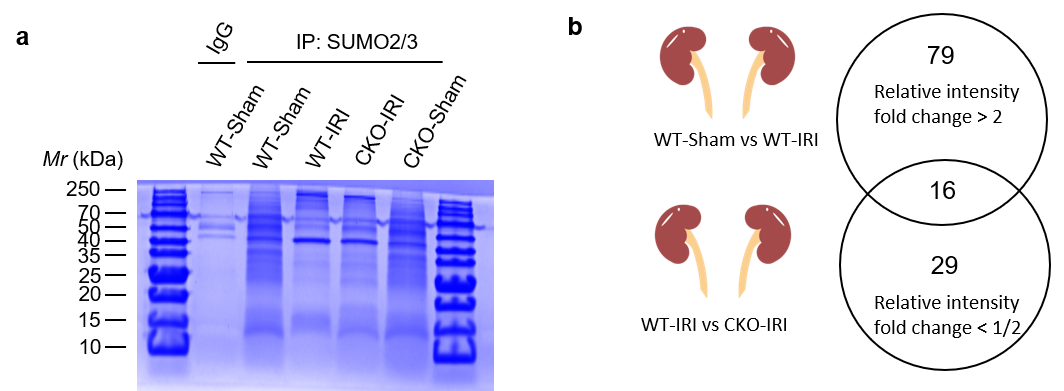


# **Figure S11 Identification of SUMO2/3-modified proteins in renal IRI.**

(a) Coomassie blue staining of SUMO2/3-conjugated proteins immunoprecipitated from kidney tissues of wild-type (WT) and Senp3 conditional knockout (CKO) mice following ischemia-reperfusion injury (IRI). (b) Schematic workflow of liquid chromatography-mass spectrometry (LC-MS) analysis and bioinformatic screening for SUMO2/3 target candidates.


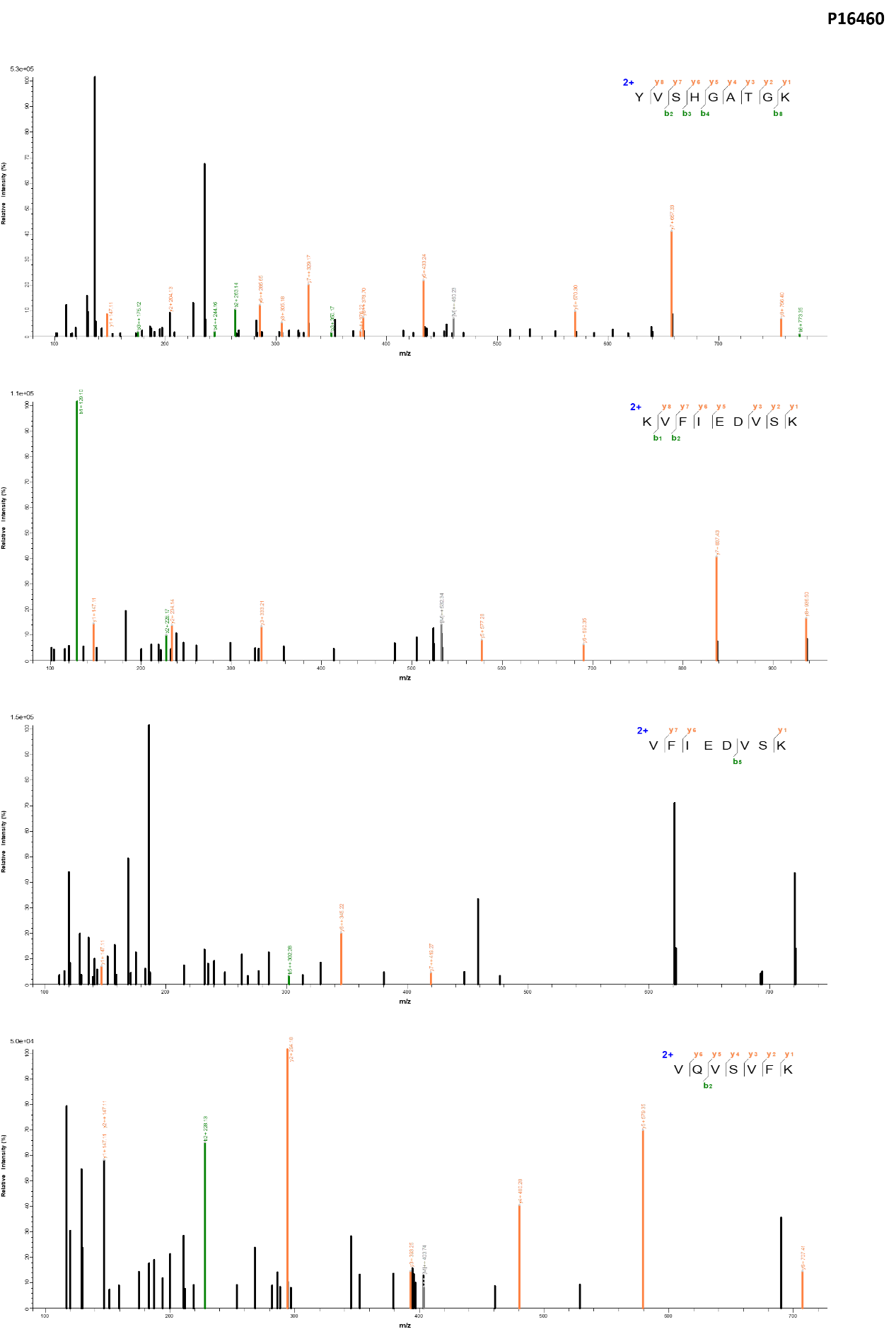


# **Figure S12 Secondary Mass Spectrometry of ASS1 (Protein ID: P16460).**


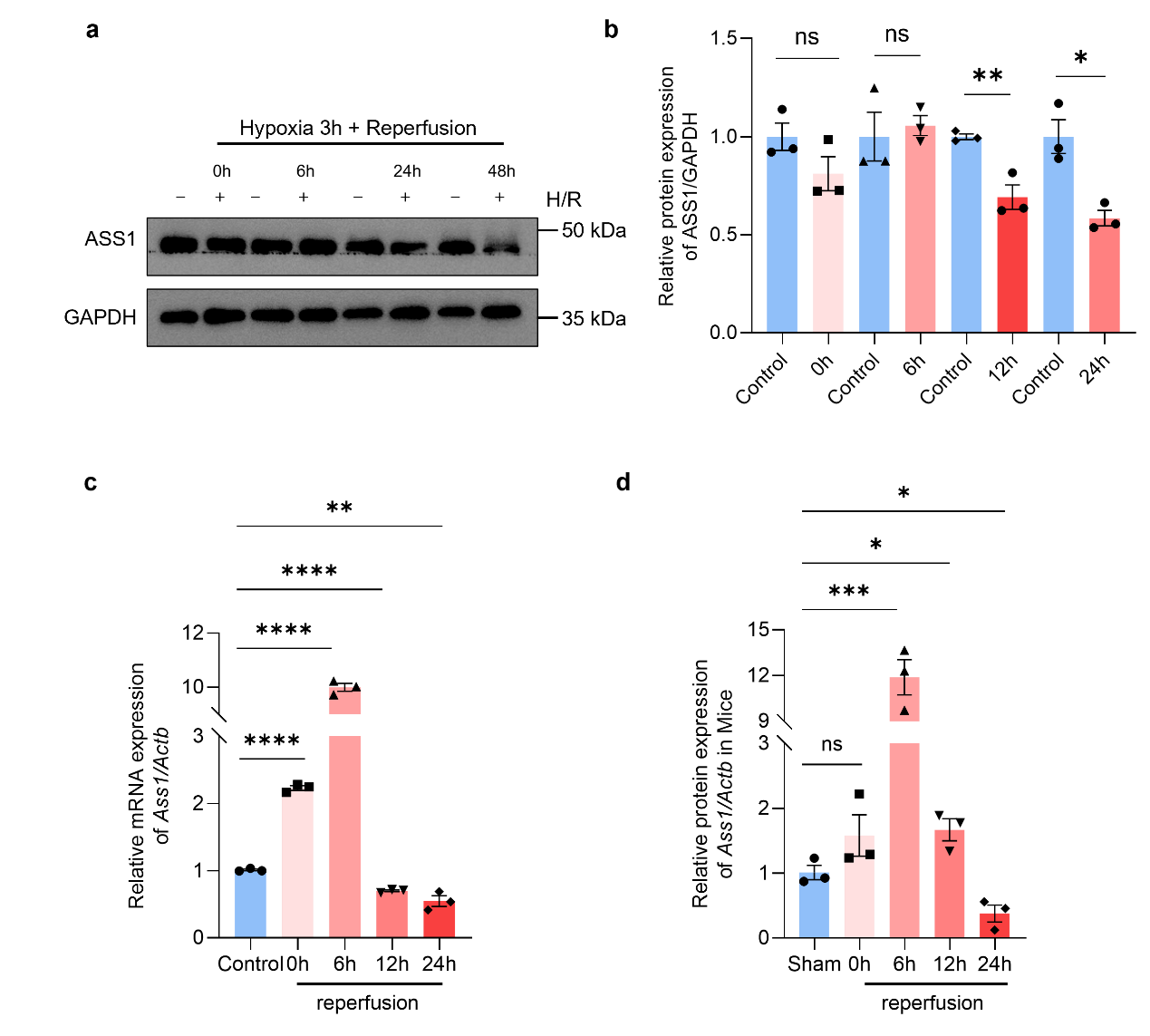


# **Figure S13 Dynamic changes in ASS1 expression following H/R.**

(a-b) Western blot analysis and quantification showing progressive decrease in ASS1 protein levels during reoxygenation in TCMK1 cells. (c-d) RT-qPCR analysis reveals biphasic transcriptional regulation of *Ass1* in TCMK1 cells (c) and mouse kidney tissues (d) after IRI or H/R, characterized by an initial increase followed by subsequent downregulation. Data: mean ±SEM (*n*=3). ns, not significance. *p <0.05, **p <0.01, ***p <0.001, ****p <0.0001.


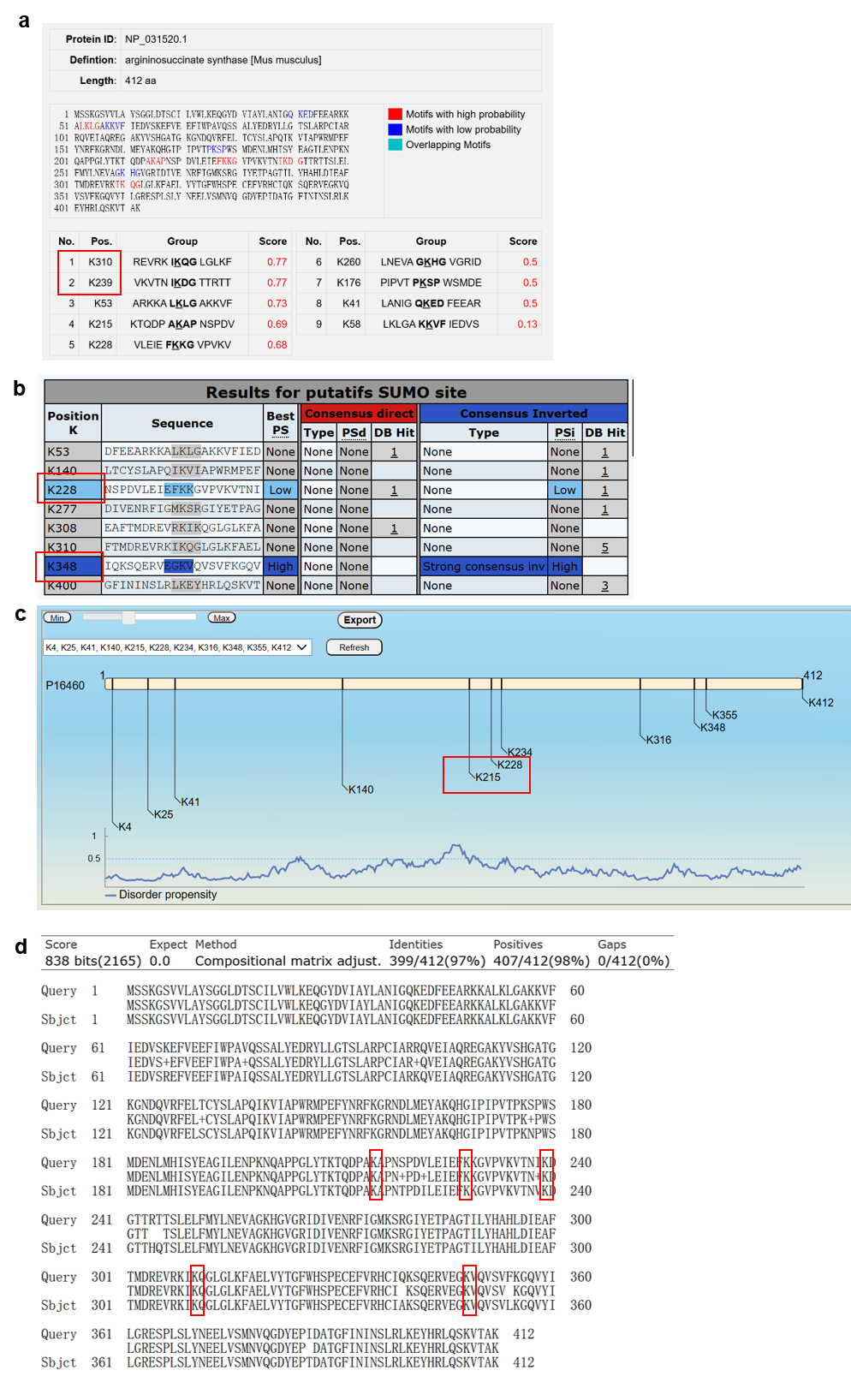


# **Figure S14 Computational prediction and cross-species conservation of ASS1 SUMOylation sites**

(a-c) In silico prediction of SUMO2/3 conjugation sites on ASS1 using three independent algorithms: (a) SUMOplot (Abcepta), (b) JASSA, and (c) GPS-SUMO. (d) Sequence alignment of mouse (top) and human (bottom) ASS1 showing evolutionary conservation of 19 experimentally reported SUMOylation sites (K58, K101, K112, K121, K155, K165, K176, K199, K209, K215, K228, K234, K239, K260, K310, K340, K355, K400, K408). Red asterisks denote validated sites from literature.


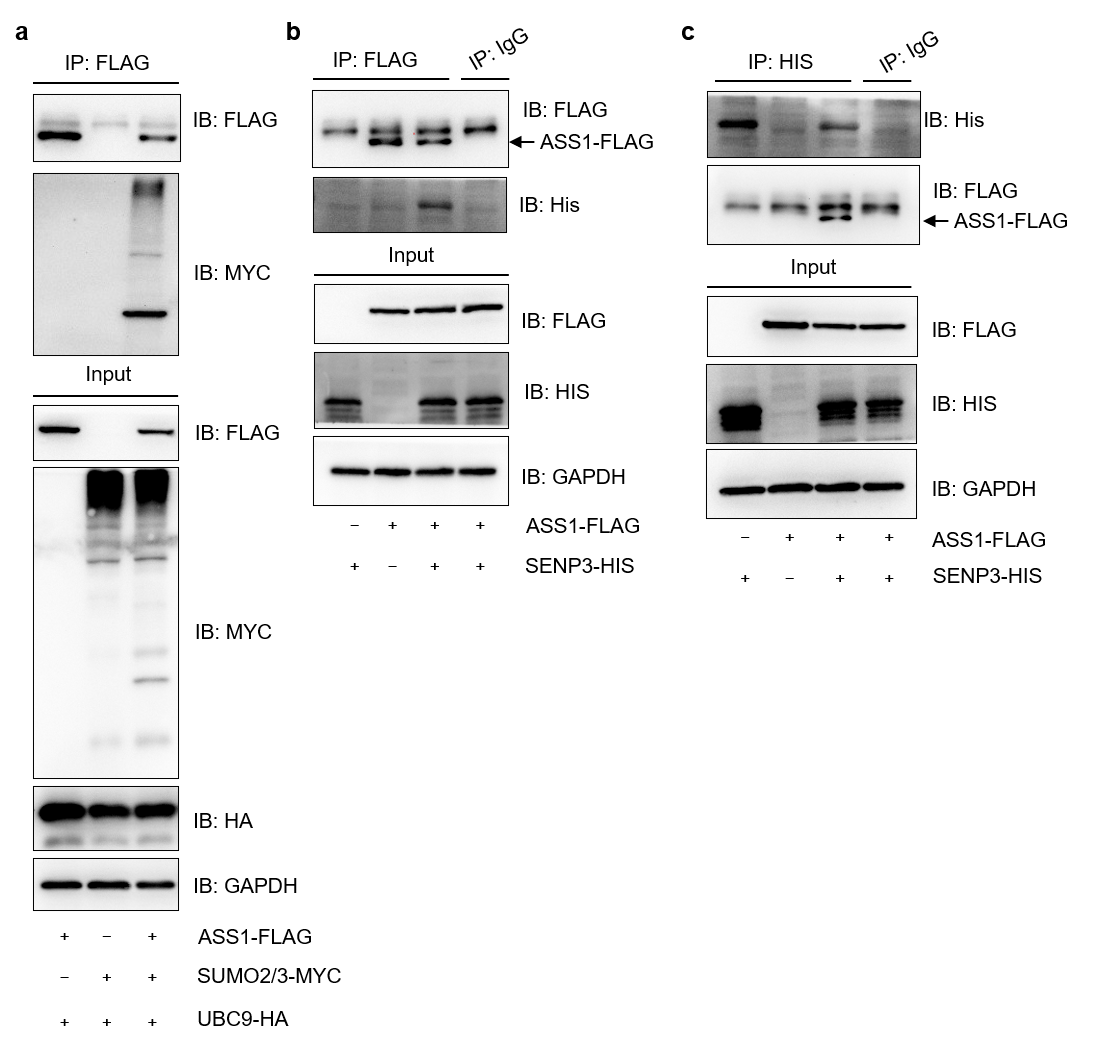


# **Figure S15 Exogenous interactions between ASS1, SUMO2/3, and SENP3.**

(a) Co-immunoprecipitation assay demonstrating interaction between FLAG-ASS1 and MYC-SUMO2/3 in HEK293T cells co-transfected with HA-UBC9 (essential E2 conjugating enzyme for SUMOylation). (b-c) Interaction studies between FLAG-ASS1 and HIS-SENP3 in transfected HEK293T cells. All experiments were performed in biological triplicates.


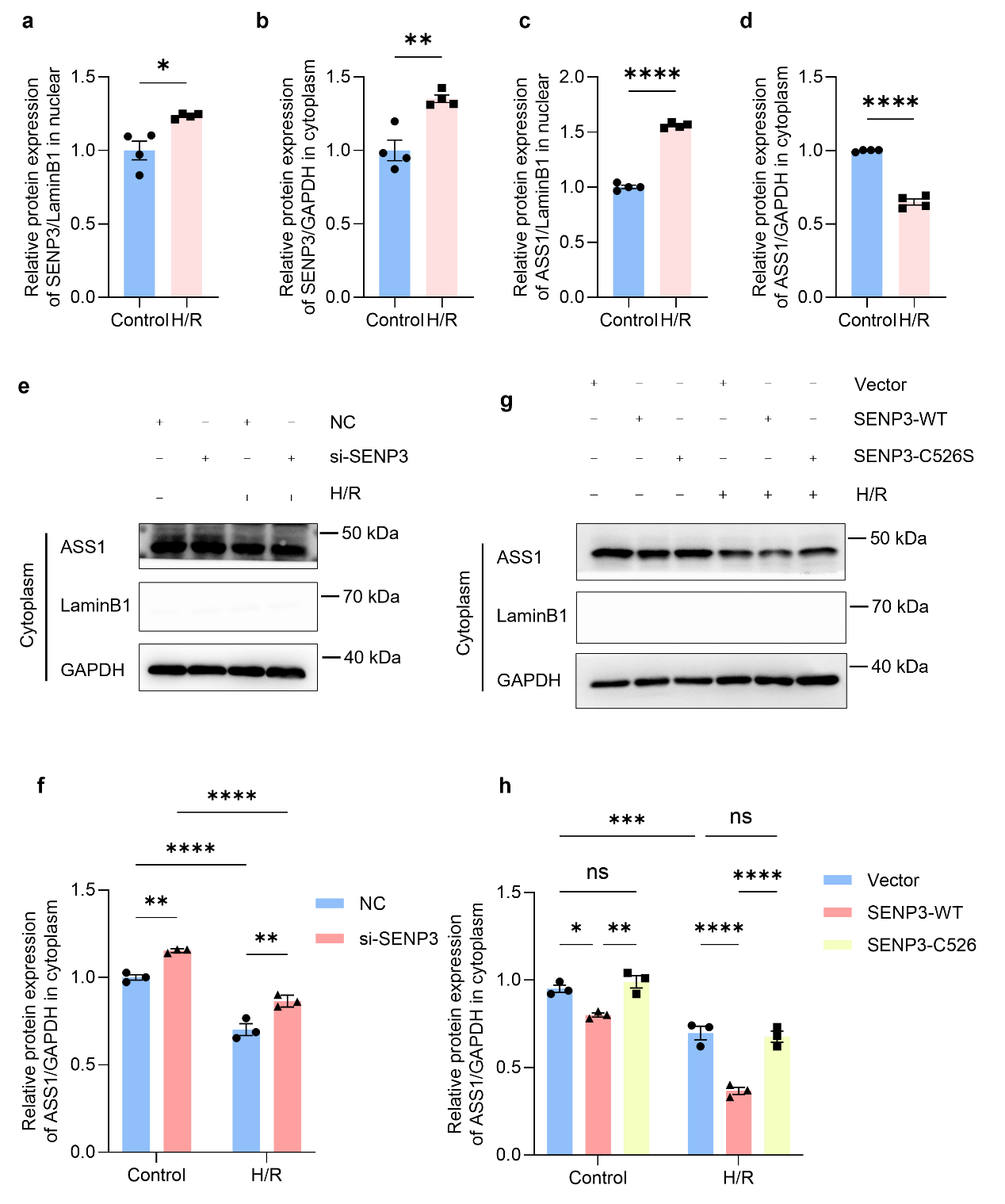


# **Figure S16 Subcellular localization of ASS1 and SENP3 in TCMK1 cells under H/R conditions.**

(a-d) Quantitative analysis of ASS1 and SENP3 distribution in nuclear and cytoplasmic fractions. (e-f) Cytoplasmic ASS1 levels in TCMK1 cells subjected to H/R with or without SENP3 knockdown. (g-h) Cytoplasmic ASS1 levels in TCMK1 cells under H/R conditions with or without functional SENP3 catalytic activity. Data represent mean ± SEM (*n=3*); ns, not significant; **p<0.01, ***p<0.001, ****p<0.0001.


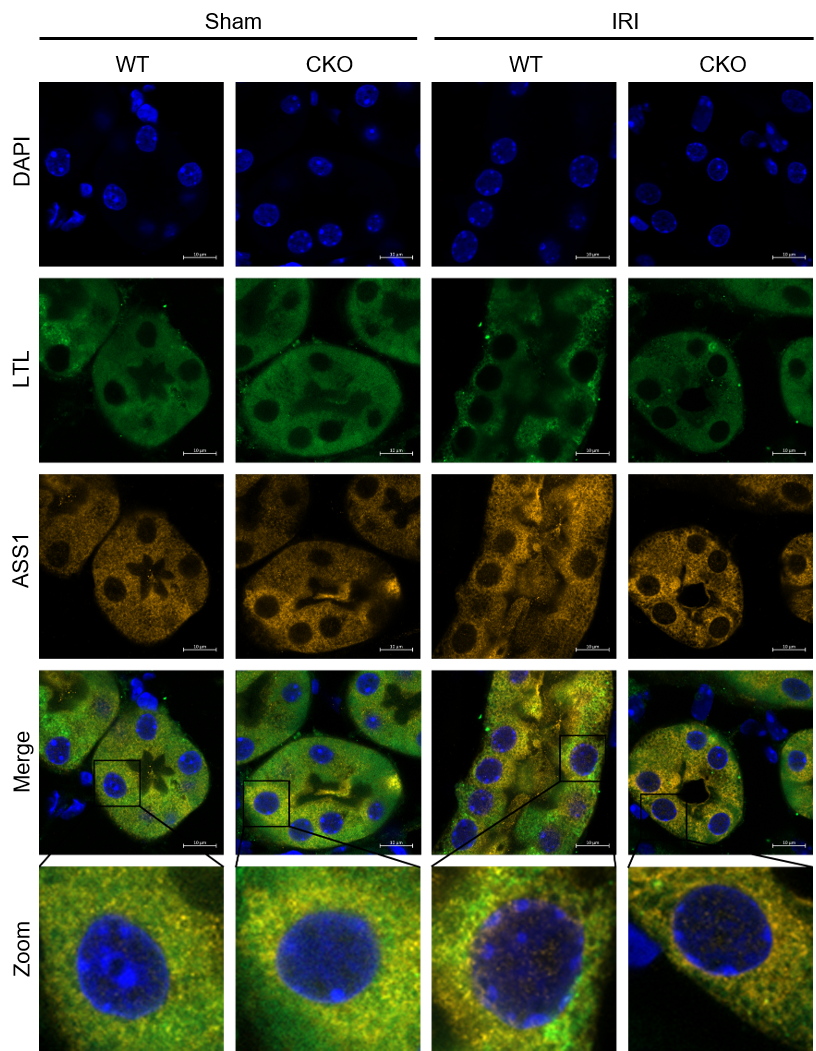


# **Figure S17 The ASS1 accumulated in nuclear after IRI in PTECs.**

Scale bar, 10μm.


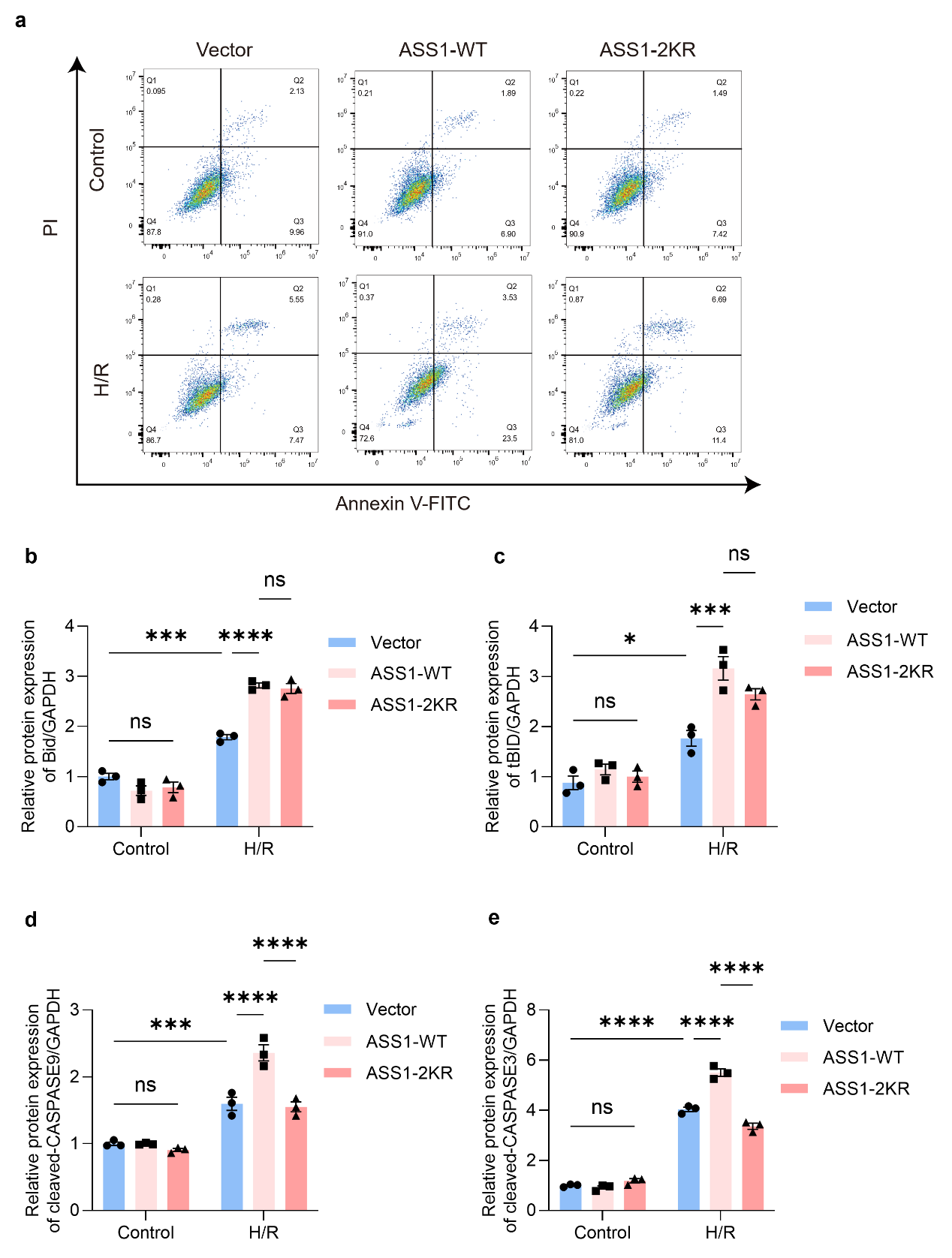


# **Figure S18 SUMOylation-deficient ASS1 attenuates H/R-induced apoptosis.**

(a) Flow cytometric analysis (Annexin V-FITC/PI) of apoptosis in TCMK1 cells transfected with ASS1-WT or the SUMOylation-deficient mutant ASS1-2KR following H/R treatment. (b-e) Quantitative analysis of apoptosis-related markers confirmed that wild-type ASS1 promotes apoptosis more effectively than the ASS1-2KR mutant. Data represent mean ± SEM (*n=3*); ns, not significant; *p<0.05, ***p<0.001, ****p<0.0001.


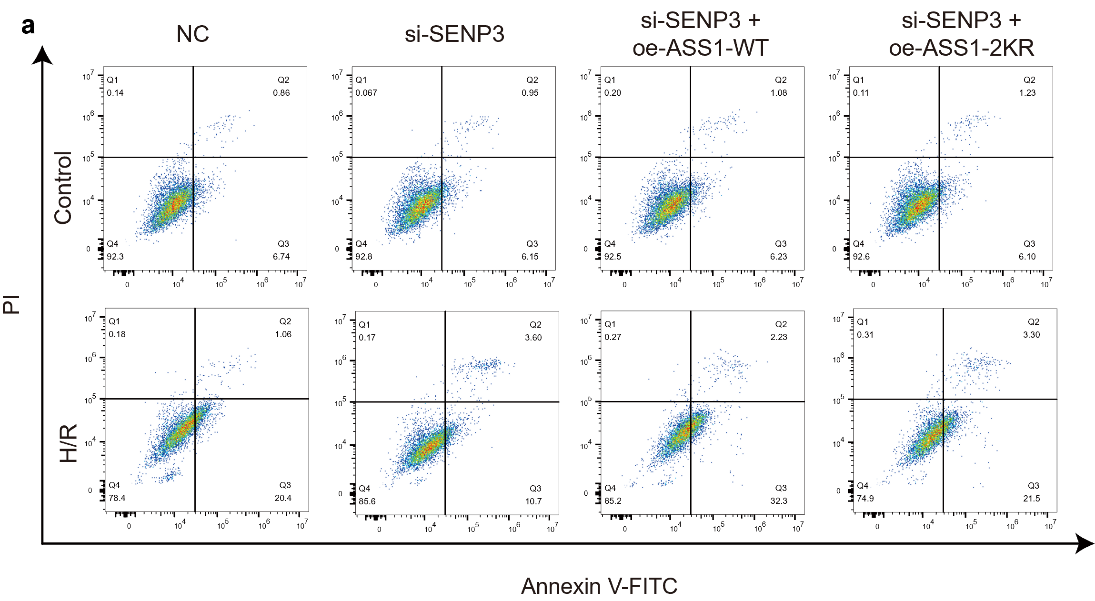


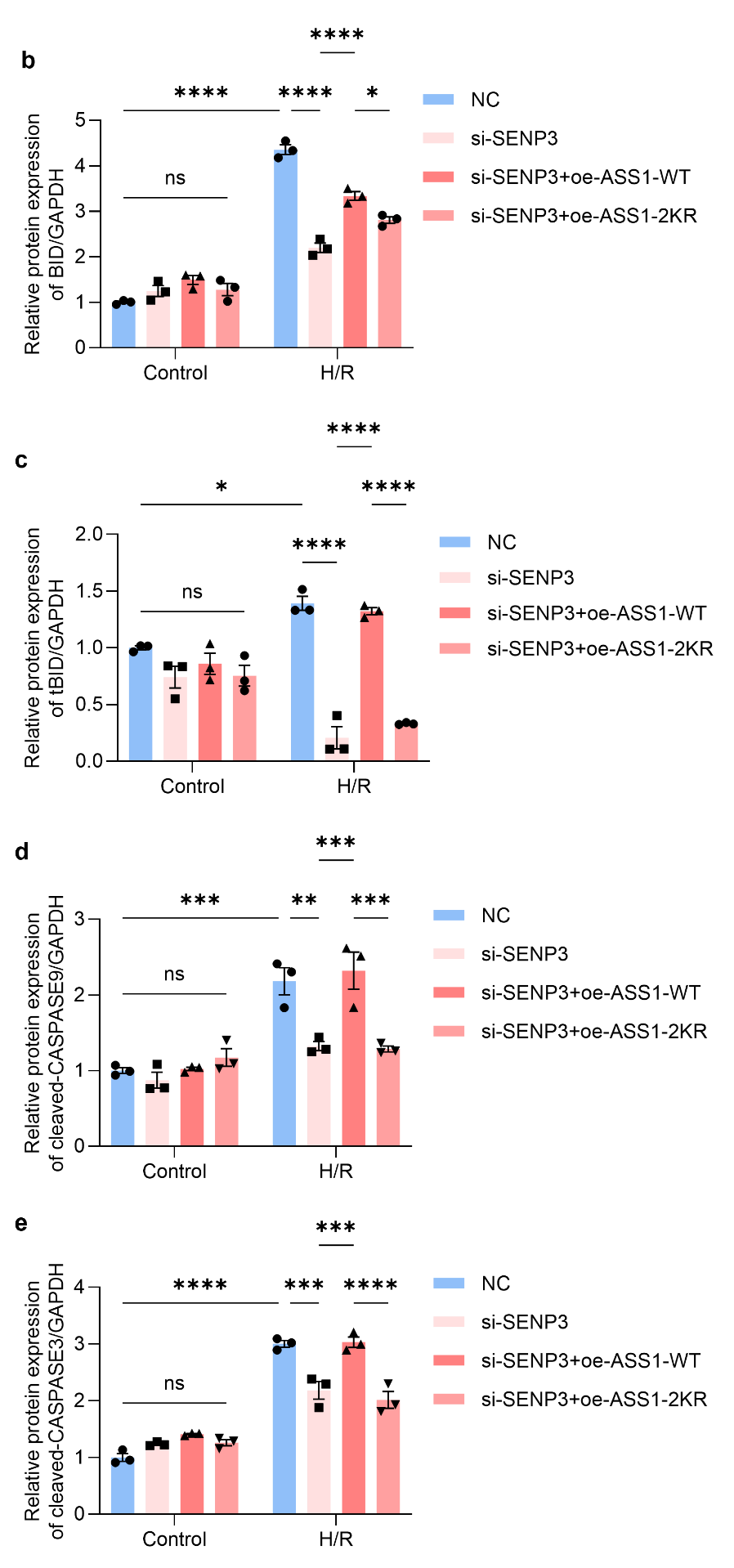


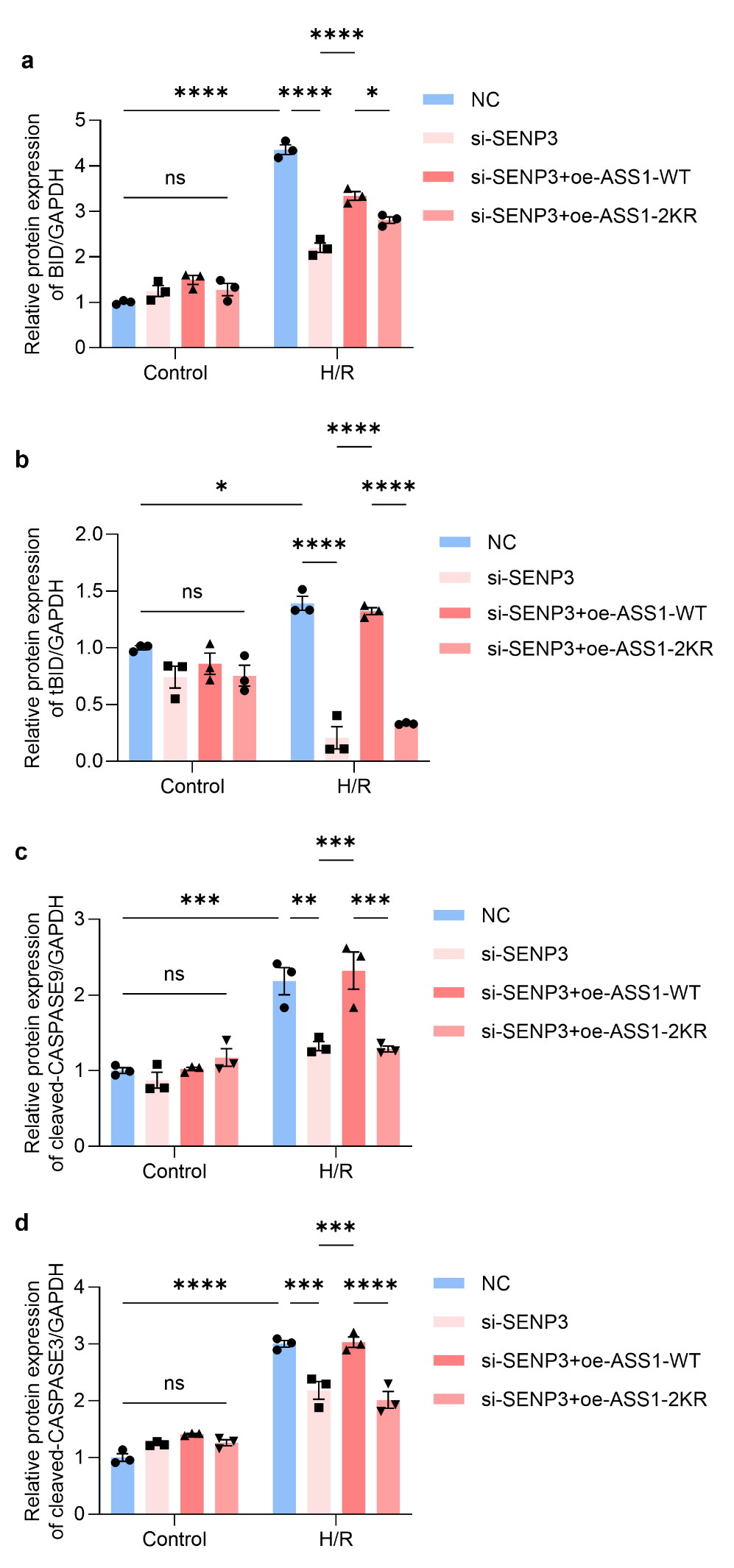


# **Figure S19 ASS1 deSUMOylation is required for the pro-apoptotic function of SENP3 under H/R conditions.**

(a-e) TCMK1 cells were co-transfected with SENP3 siRNA and either wild-type ASS1 (ASS1-WT) or the SUMOylation-deficient mutant (ASS1-2KR) for 24 hours, followed by H/R treatment. Flow cytometric analysis (Annexin V-FITC/PI) and immunoblotting of apoptosis-related proteins demonstrated that ASS1-WT, but not ASS1-2KR, restored apoptosis in SENP3-deficient cells. Data: mean ±SEM (*n*=3). ns, not significance. *p <0.05, **p <0.01, ***p <0.001, ****p <0.0001.


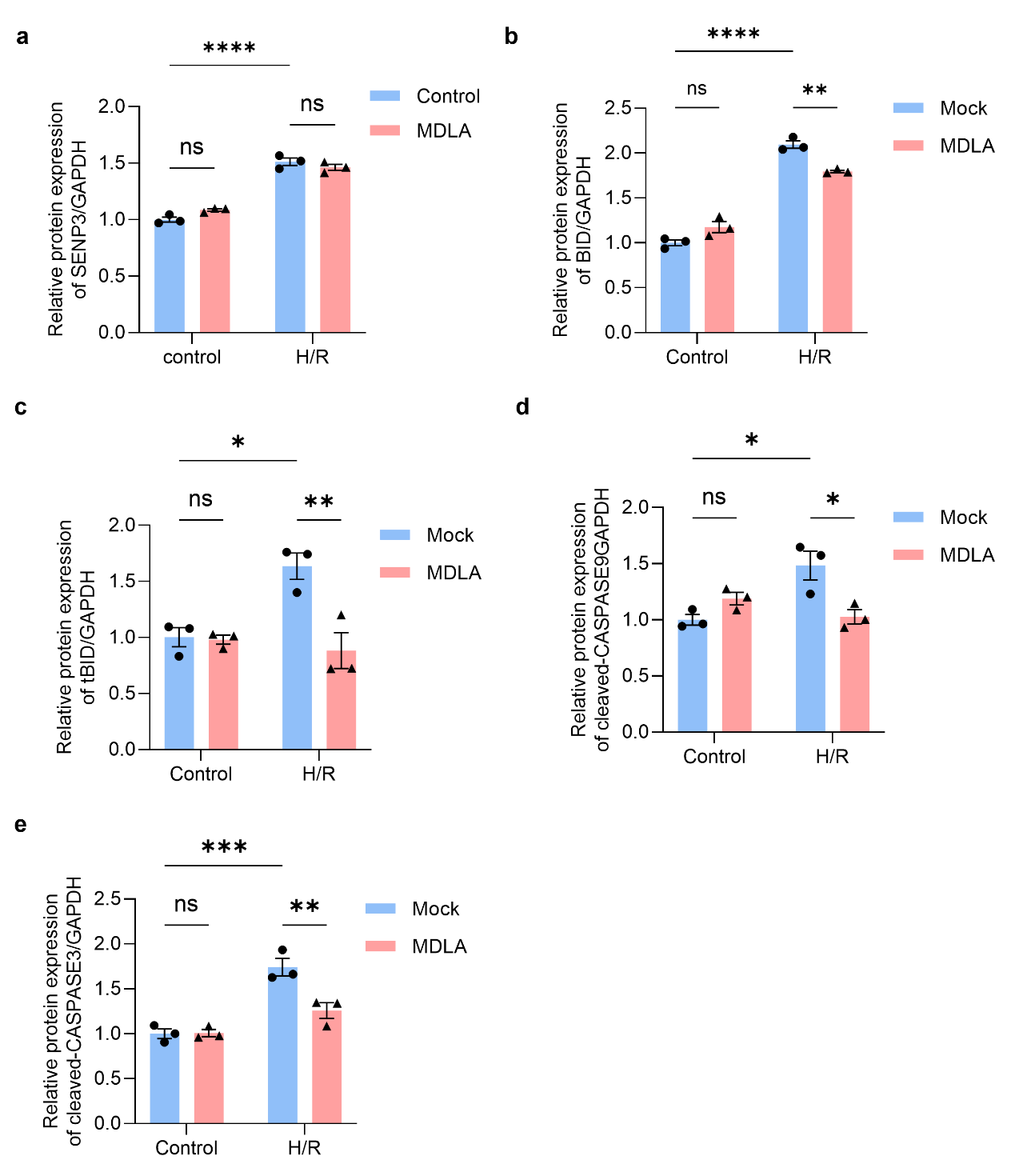


# **Figure S20 ASS1 Inhibitor MDLA attenuates H/R-induced apoptosis in TCMK1 cells.**

(a-d) Treatment with the ASS1 inhibitor MDLA did not affect SENP3 expression but significantly reduced levels of apoptosis-related proteins in TCMK1 cells subjected to H/R. Data: mean ±SEM (*n=3*). ns, no significance. *p<0.05, **p<0.01, ***p<0.001.


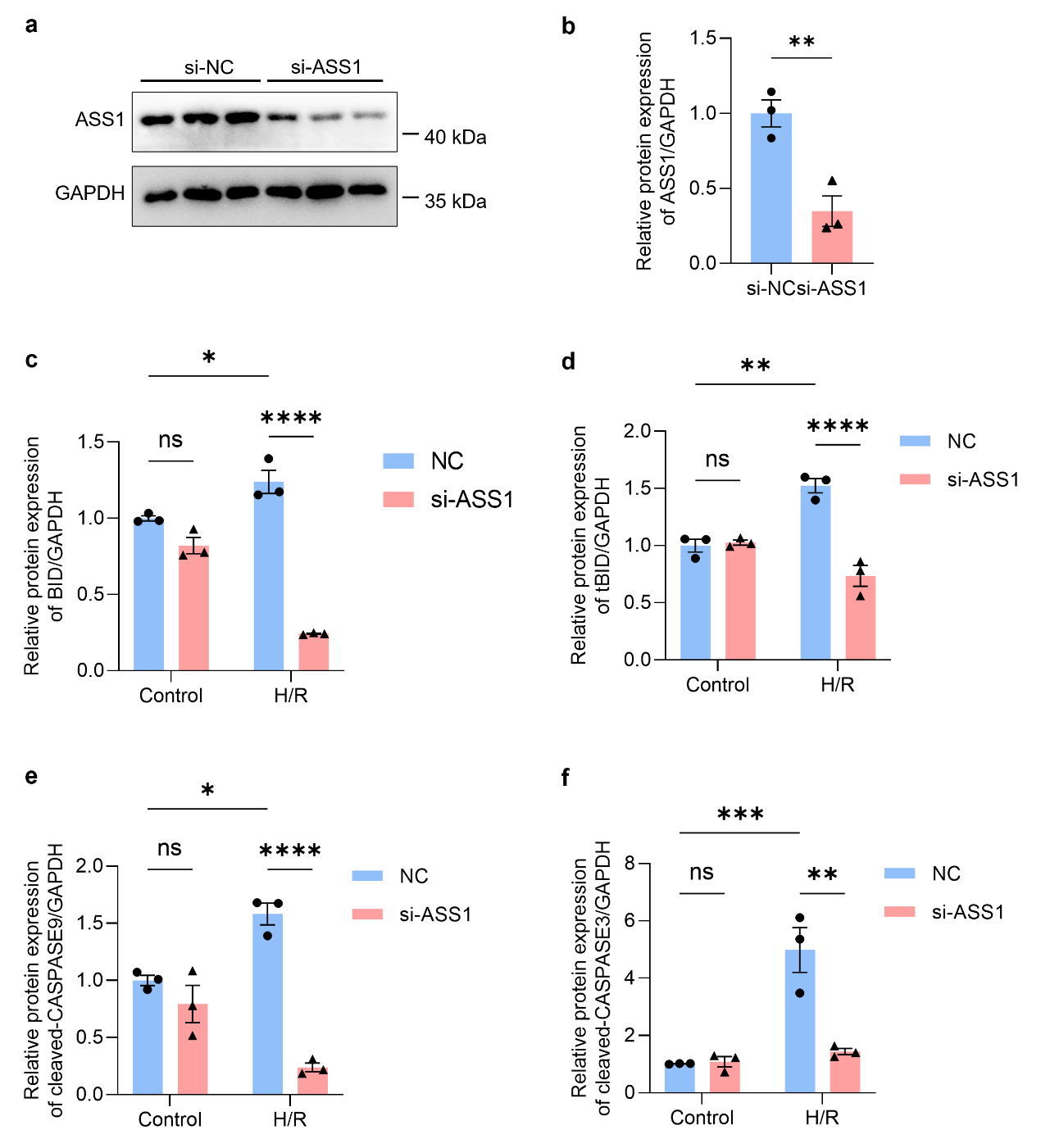


# **Figure S21 ASS1 deficiency attenuates H/R-induced apoptosis in TCMK1 cells.**

(a-b) Western blot analysis and quantification confirmed efficient knockdown of ASS1 in TCMK1 cells transfected with ASS1-targeting siRNA (si-ASS1) compared to negative control siRNA (si-NC). (c-f) ASS1 silencing significantly reduced the activation of apoptotic pathway in H/R-treated TCMK1 cells. Data represent mean ± SEM (*n=3*). ns, no significance. **p < 0.01.


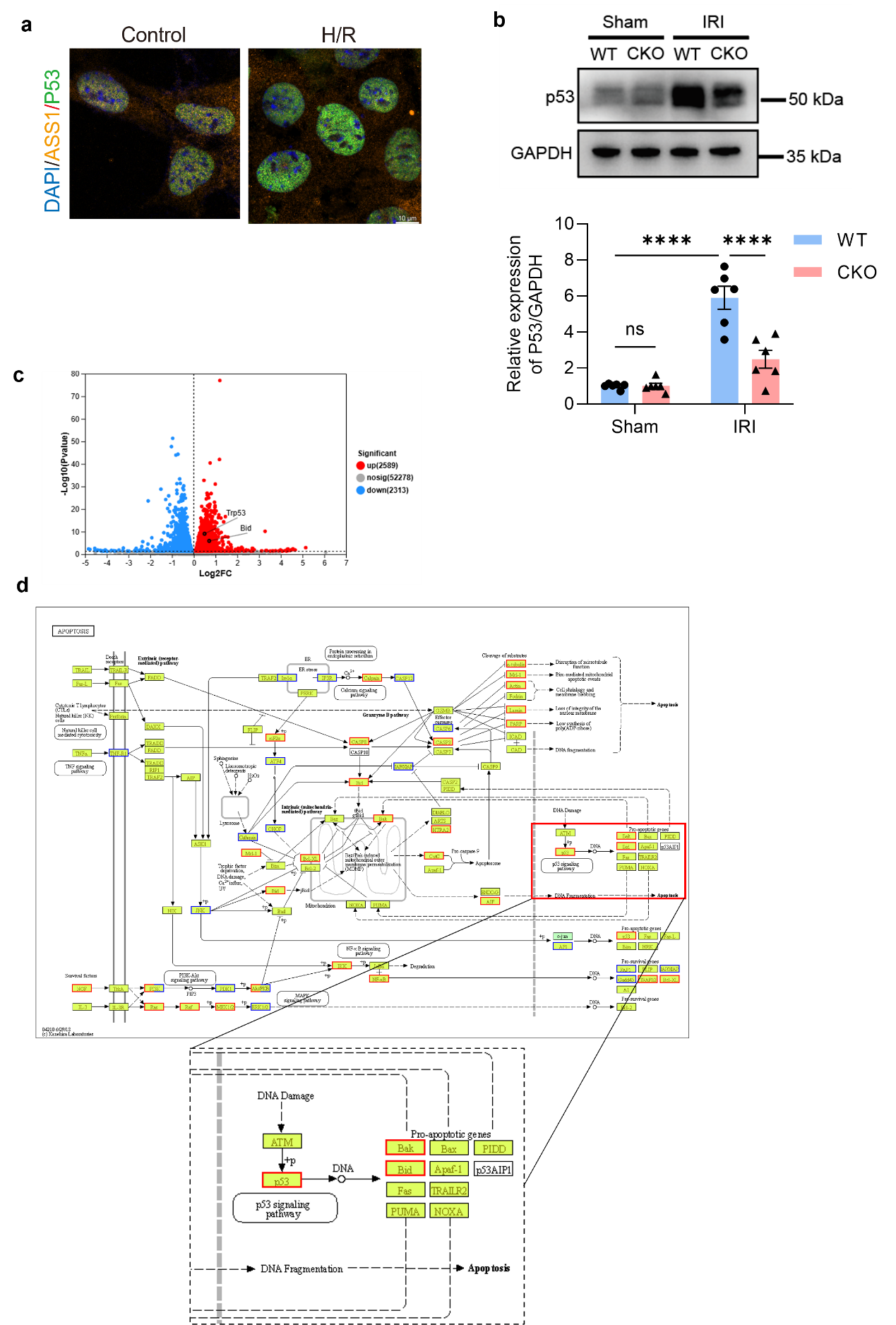


# **Figure S22 Hypoxia/reoxygenation (H/R) induces Trp53-mediated transcriptional activation of Bid.**

(a) Co-localization of ASS1 and p53 in TCMK1 cells after H/R. (b) SENP3 deficiency reduces p53 expression in renal tissues following IRI. Data represent mean ± SEM (*n=3*). ns, no significance. ****p < 0.0001. (b) Volcano plot showing differentially expressed genes in TCMK1 mouse renal epithelial cells following H/R treatment. RNA-seq analysis was performed using Mus musculus reference genome (GRCm39). Differential expressions were determined by DESeq2 (p<0.05, *n=3*). (c) KEGG pathway enrichment analysis demonstrates significant activation of apoptosis-related genes, including *Bid*.


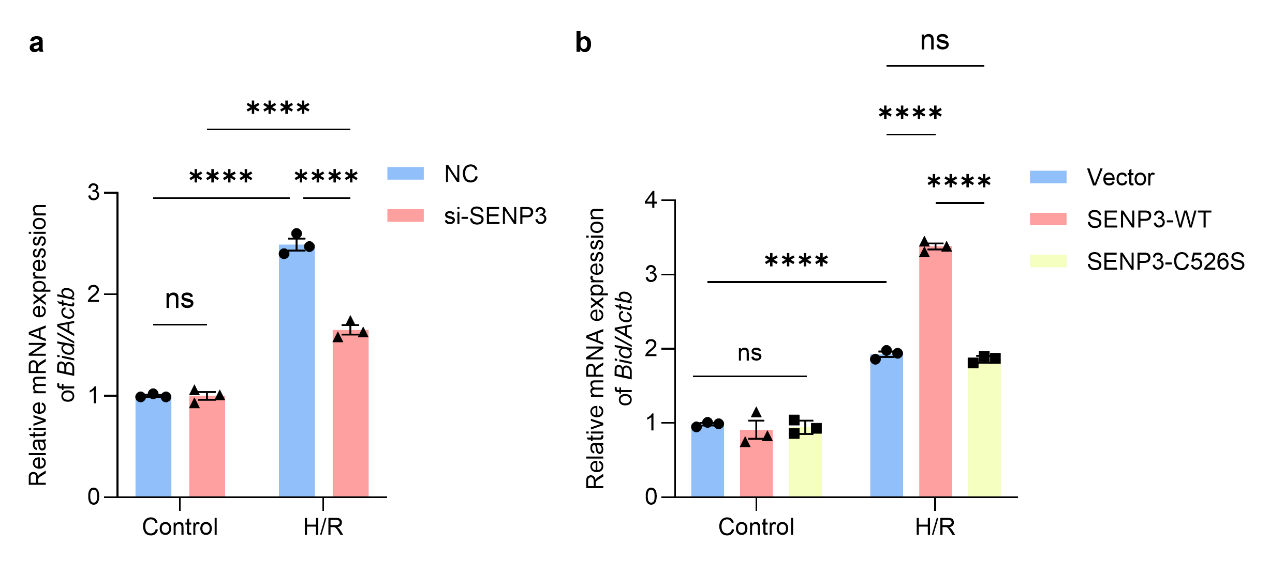


# **Figure S23 The transcription of Bid.**

(a-b) RT-qPCR analysis demonstrating SENP3 deSUMOylation-dependent regulation of Bid expression that paralleled Trp53 transcriptional activation. Data represent mean ± SEM (*n=3*). ns, no significance. ****p <0.0001.

# **Table S1 The details of primary antibodies.**

| Protein | Primary Antibody |
| --- | --- |
| SENP3 | #5591，CST |
| SUMO2/3 | ab81371, Abcam |
| ASS1 | 16210-1-AP, Proteintech |
| GAPDH | 60004-1-Ig, Proteintech |
| HA | AE105, ABclonal |
| MYC | 19C2, ABmart |
| FLAG | 20543-1-AP, Proteintech |
| HIS | 66005-1-IG, Proteintech |
| BID/tBID | S0B0408, STARTER;10988-1-AP, Proteintech |
| Cleaved CASPASE-3 | 19677-1-AP, Proteintech; 9664T, CST |
| Cleaved CASPASE-9 | 10380-1-AP, Proteintech |
| Cleaved CASPASE-8 | 66093-1-Ig, Proteintech |
| P53 | 60283-2-Ig, Proteintech |

# **Table S2 The details of primers of RT-qPCR.**

| *Gene* | Forward | Reverse |
| --- | --- | --- |
| *Senp1* | CTGGGGAGGTGACCTTAGTGA | GTGATAATCTGGACGATAGGCTG |
| *Senp2* | AAGAACAGTCTCTACAATGCTGC | CCGATTTCAGCGTAAAACCAAAG |
| *Senp3* | CCGGCCATCTTTTGATGCCT | GCGAGGTGCTTTTTGAGTAGAG |
| *Senp5* | AGCCAATGGTCACGAACCTAC | GCCATGACAAACTCGCTTTCTA |
| *Senp6* | GTTGTTTGTTTCCCTGGTTTGG | GCACTCGAATCAGTCACAGCTA |
| *Senp7* | CTATGGACGGACTTAGGACGA | CAGATGTCGAAGGCAATGAGT |
| *Actb* | GTGACGTTGACATCCGTAAAGA-3’ | GCCGGACTCATCGTACTCC-3’ |
| *Ass1* | TGTACCTGAACGAAGTTGCGG | ACCTCGGGACTTCATTCCAAT |
| *Ngal* | TGGCCCTGAGTGTCATGTG | CTCTTGTAGCTCATAGATGGTGC |
| *Kim-1* | CCTTGTGAGCACCGTGGCTA | TGTTGTCTTCAGCTCGGGAATG |

# **Table S3 The characteristics of candidate proteins.**

|  | Proteintein IDs | Description | Fold Change of Intensity | | Mouse Gene | Human Gene | Human Homologous Protein | Biological Process | Protein Expression | | | |
| --- | --- | --- | --- | --- | --- | --- | --- | --- | --- | --- | --- | --- |
|  |  |  | WT-sham/WT-IRI | WT-IRI/CKO-IRI |  |  |  |  | kidney | cells in Tubules | promixal tubulus (cell body) | proximal tubules (microvilli) |
| 1 | P01864 | Ig gamma-2A chain C region secreted form OS=Mus musculus OX=10090 PE=1 SV=1 | 165.50 | 0.15 | Ighg2a | IGHG2 | P01859 · IGHG2_HUMAN | Adaptive immunity, Immunity | medium | medium | - | - |
| 2 | P07724 | Albumin OS=Mus musculus OX=10090 GN=Alb PE=1 SV=3 | 168.52 | 0.36 | Alb | ALB | P02768 · ALBU_HUMAN | Calcium, Copper, Lipid-binding, Metal-binding, Zinc | medium | - | low | medium |
| 3 | P16460 | Argininosuccinate synthase OS=Mus musculus OX=10090 GN=Ass1 PE=1 SV=1 | 323.26 | 0.16 | Ass1 | ASS1 | P00966 · ASSY_HUMAN | Amino-acid biosynthesis, Arginine biosynthesis, Urea cycle | high | - | high | not detected |
| 4 | P17427 | AP-2 complex subunit alpha-2 OS=Mus musculus OX=10090 GN=Ap2a2 PE=1 SV=2 | 5.03 | 0.04 | Ap2a2 | AP2A2 | O94973 · AP2A2_HUMAN | Endocytosis, Protein transport, Transport | medium | medium | - | - |
| 5 | P50516 | V-type proton ATPase catalytic subunit A OS=Mus musculus OX=10090 GN=Atp6v1a PE=1 SV=2 | 394.24 | 0.42 | Atp6v1a | ATP6V1A | P38606 · VATA_HUMAN | Hydrogen ion transport, Ion transport, Transport | high | medium | - | - |
| 6 | P57780 | Alpha-actinin-4 OS=Mus musculus OX=10090 GN=Actn4 PE=1 SV=1 | 2.32 | 0.08 | Actn4 | ACTN4 | O43707 · ACTN4_HUMAN | Protein transport, Transport | low | medium | - | - |
| 7 | P63017 | Heat shock cognate 71 kDa protein OS=Mus musculus OX=10090 GN=Hspa8 PE=1 SV=1 | 558.12 | 0.14 | Hspa8 | HSPA8 | P11142 · HSP7C_HUMAN | Autophagy, Host-virus interaction, mRNA processing, mRNA splicing, Stress response, Transcription, Transcription regulation | low | low | - | - |
| 8 | Q60604 | Adseverin OS=Mus musculus OX=10090 GN=Scin PE=1 SV=3 | 4.42 | 0.26 | Scin | SCIN | Q9Y6U3 · SCIN_HUMAN | - | high | - | not detected | not detected |
| 9 | Q62468 | Villin-1 OS=Mus musculus OX=10090 GN=Vil1 PE=1 SV=3 | 6.13 | 0.49 | Vil1 | VIL1 | P09327 · VILI_HUMAN | Apoptosis | high | - | not detected | high |
| 10 | Q64331 | Unconventional myosin-VI OS=Mus musculus OX=10090 GN=Myo6 PE=1 SV=1 | 2.26 | 0.15 | Myo6 | MYO6 | Q9UM54 · MYO6_HUMAN | Endocytosis, Hearing, Protein transport, Transport | high | high | - | - |
| 11 | Q68FD5 | Clathrin heavy chain 1 OS=Mus musculus OX=10090 GN=Cltc PE=1 SV=3 | 2.78 | 0.33 | Cltc | CLTC | Q00610 · CLH1_HUMAN | Autophagy, Cell cycle, Cell division, Mitosis | medium | medium | - | - |
| 12 | Q7TMK9 | Heterogeneous nuclear ribonucleoprotein Q OS=Mus musculus OX=10090 GN=Syncrip PE=1 SV=2 | 6.91 | 0.04 | Syncrip | SYNCRIP | O60506 · HNRPQ_HUMAN | Host-virus interaction, mRNA processing, mRNA splicing, Translation regulation | medium | medium | - | - |
| 13 | Q8VDD5 | Myosin-9 OS=Mus musculus OX=10090 GN=Myh9 PE=1 SV=4 | 5.38 | 0.27 | Myh9 | MYH9 | P35579 · MYH9_HUMAN | Cell adhesion, Cell shape | high | low | - | - |
| 14 | Q8VEK3 | Heterogeneous nuclear ribonucleoprotein U OS=Mus musculus OX=10090 GN=Hnrnpu PE=1 SV=1 | 3.98 | 0.38 | Hnrnpu | HNRNPU | Q00839 · HNRPU_HUMAN | Biological rhythms, Cell cycle, Cell division, Differentiation, Host-virus interaction, Mitosis, mRNA processing, mRNA splicing, Transcription, Transcription regulation | high | high | - | - |
| 15 | Q9DBG3 | AP-2 complex subunit beta OS=Mus musculus OX=10090 GN=Ap2b1 PE=1 SV=1 | 14.42 | 0.01 | Ap2b1 | AP2B1 | P63010 · AP2B1_HUMAN | Endocytosis, Protein transport, Transport | medium | medium | - | - |
| 16 | Q9WTI7 | Unconventional myosin-Ic OS=Mus musculus OX=10090 GN=Myo1c PE=1 SV=2 | 4.31 | 0.21 | Myo1c | MYO1C | O00159 · MYO1C_HUMAN | - | medium | medium | - | - |

# **The original figures of Western Blot.**


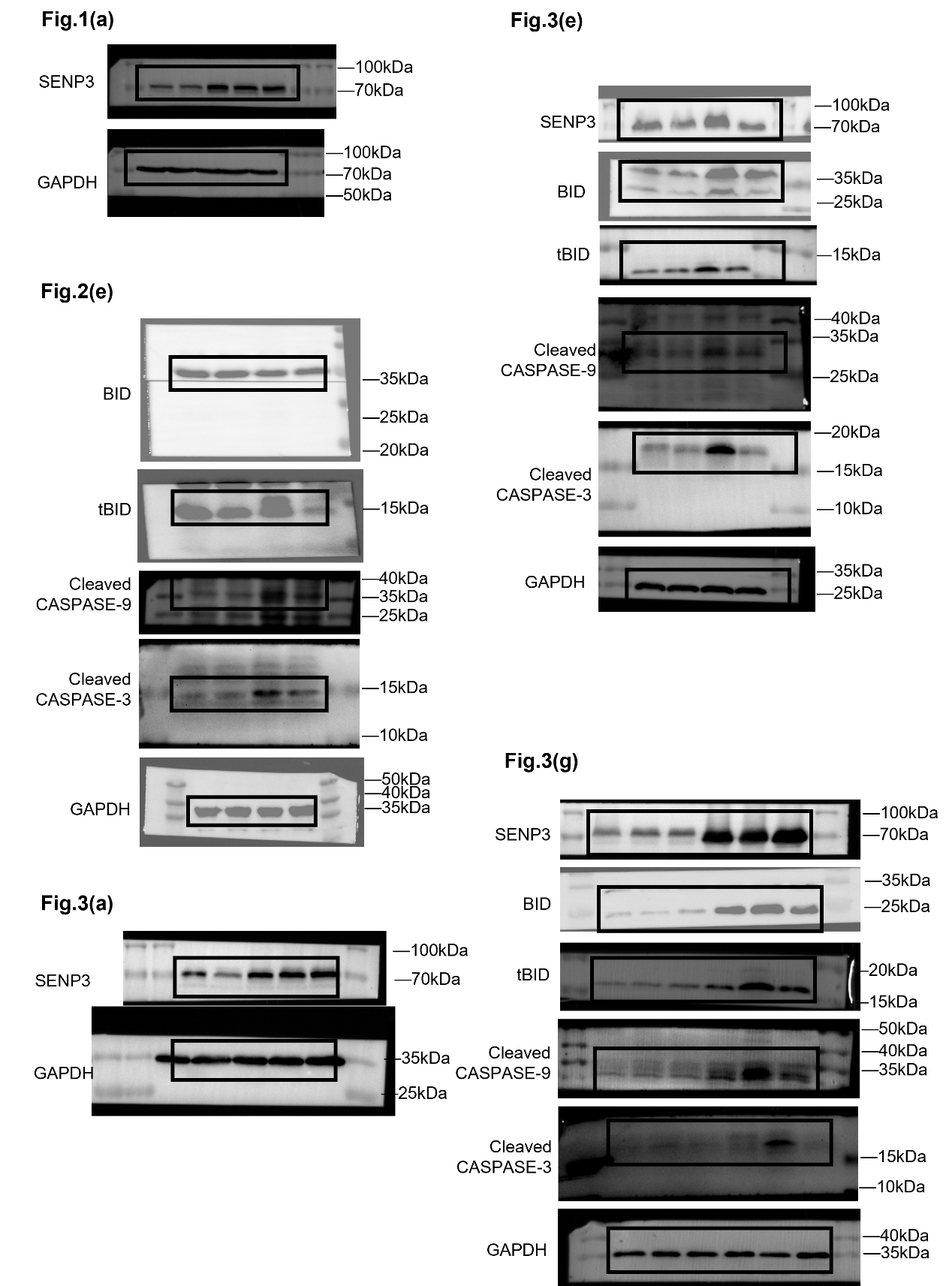


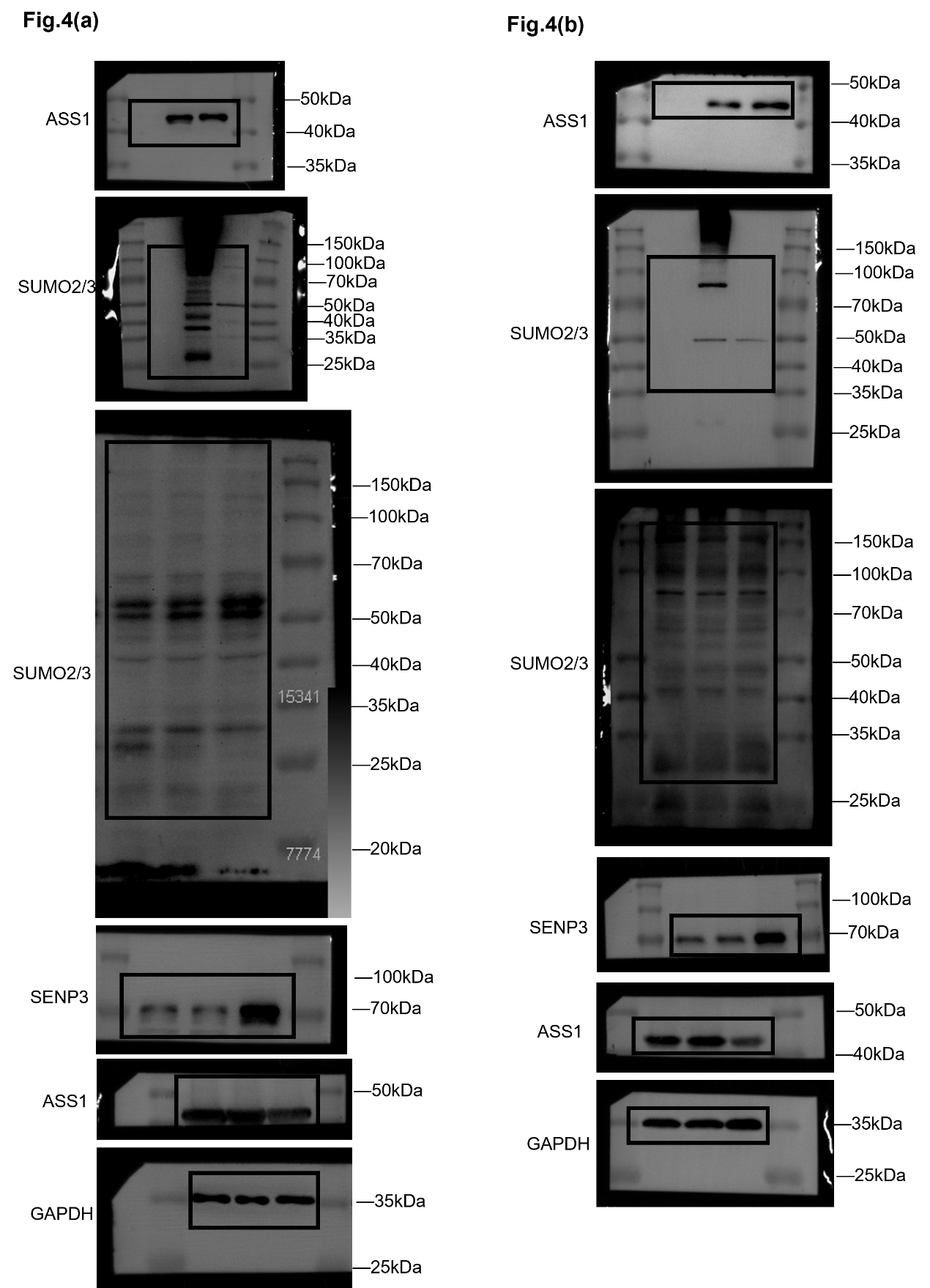

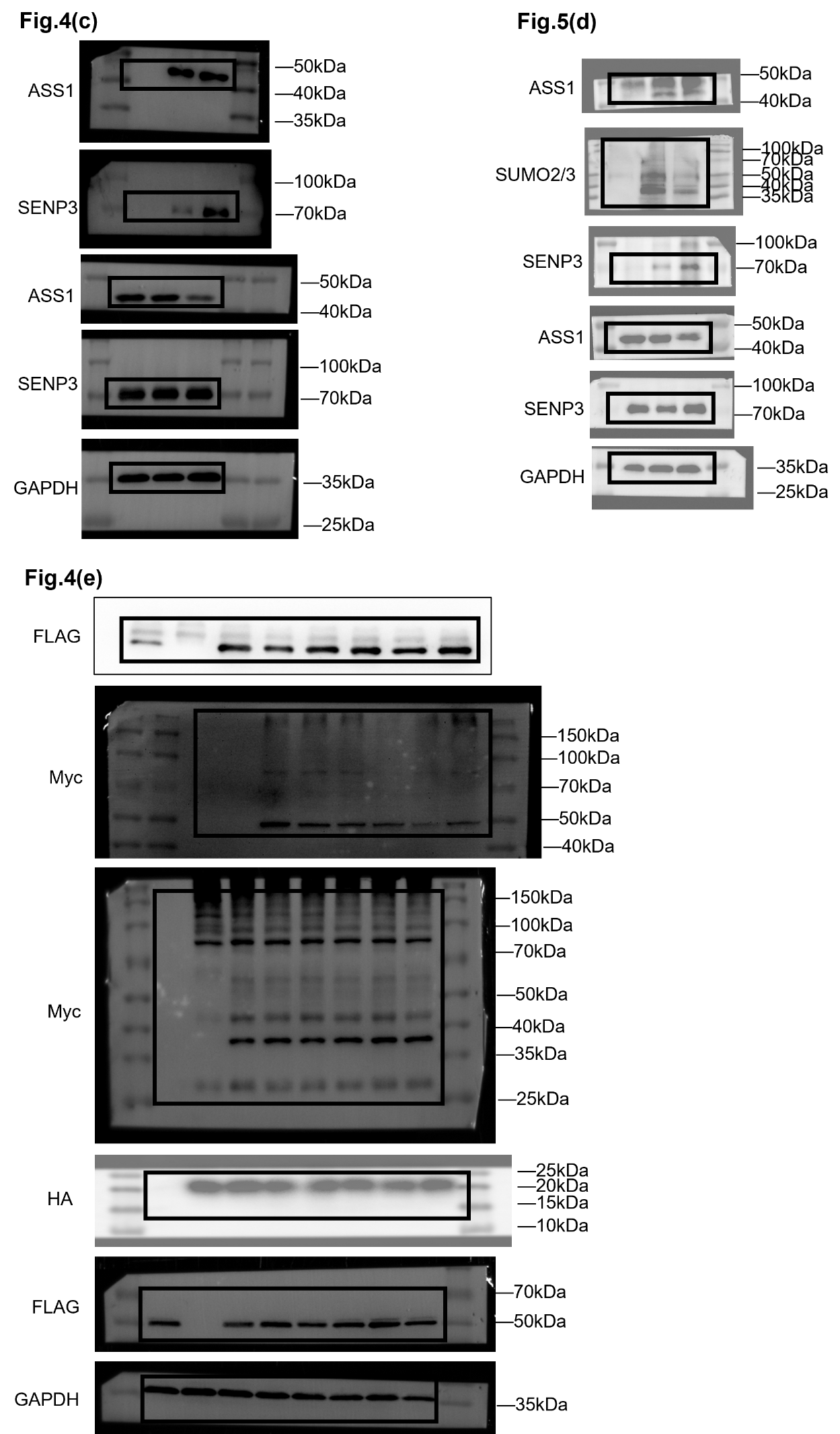

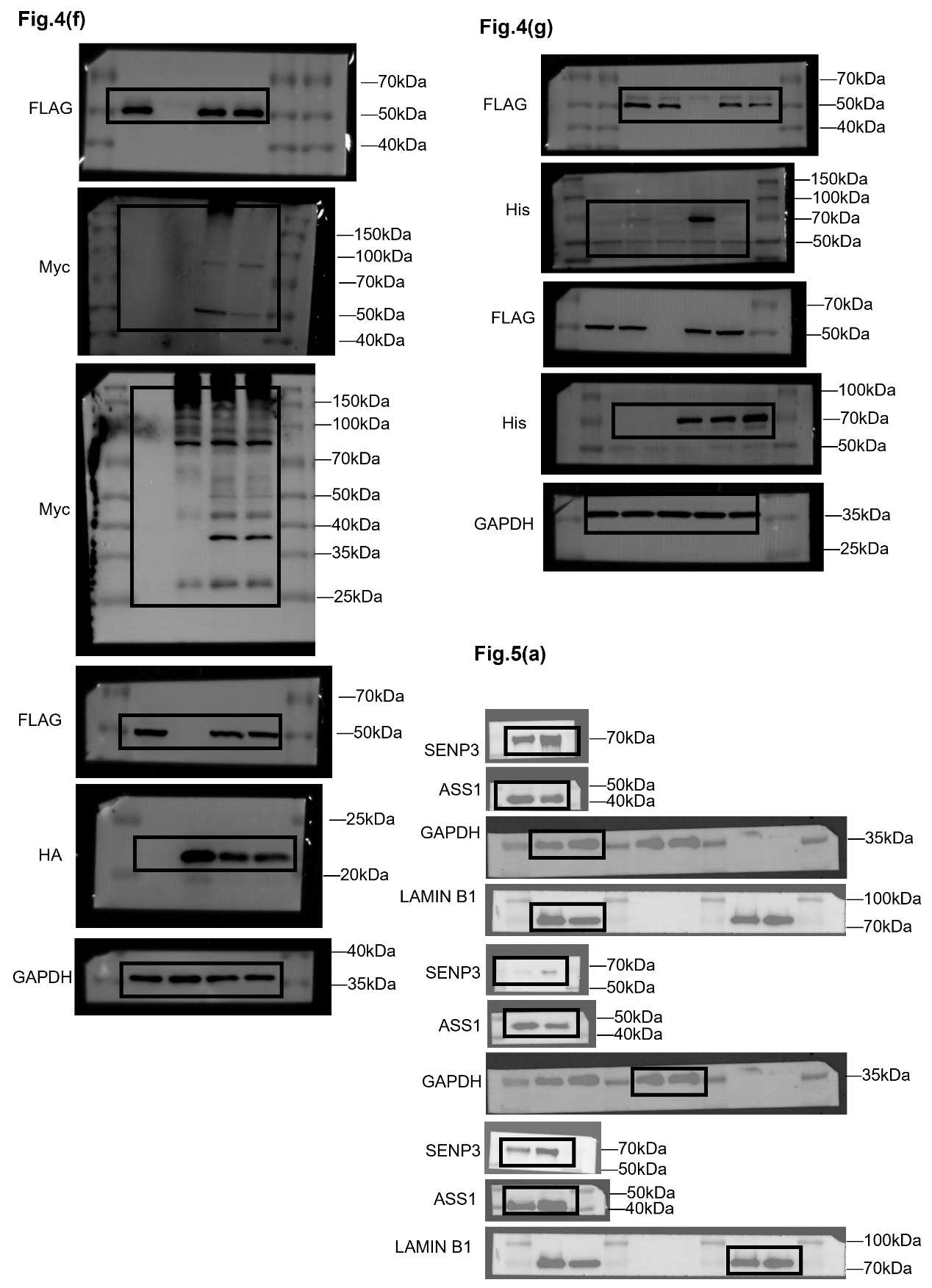

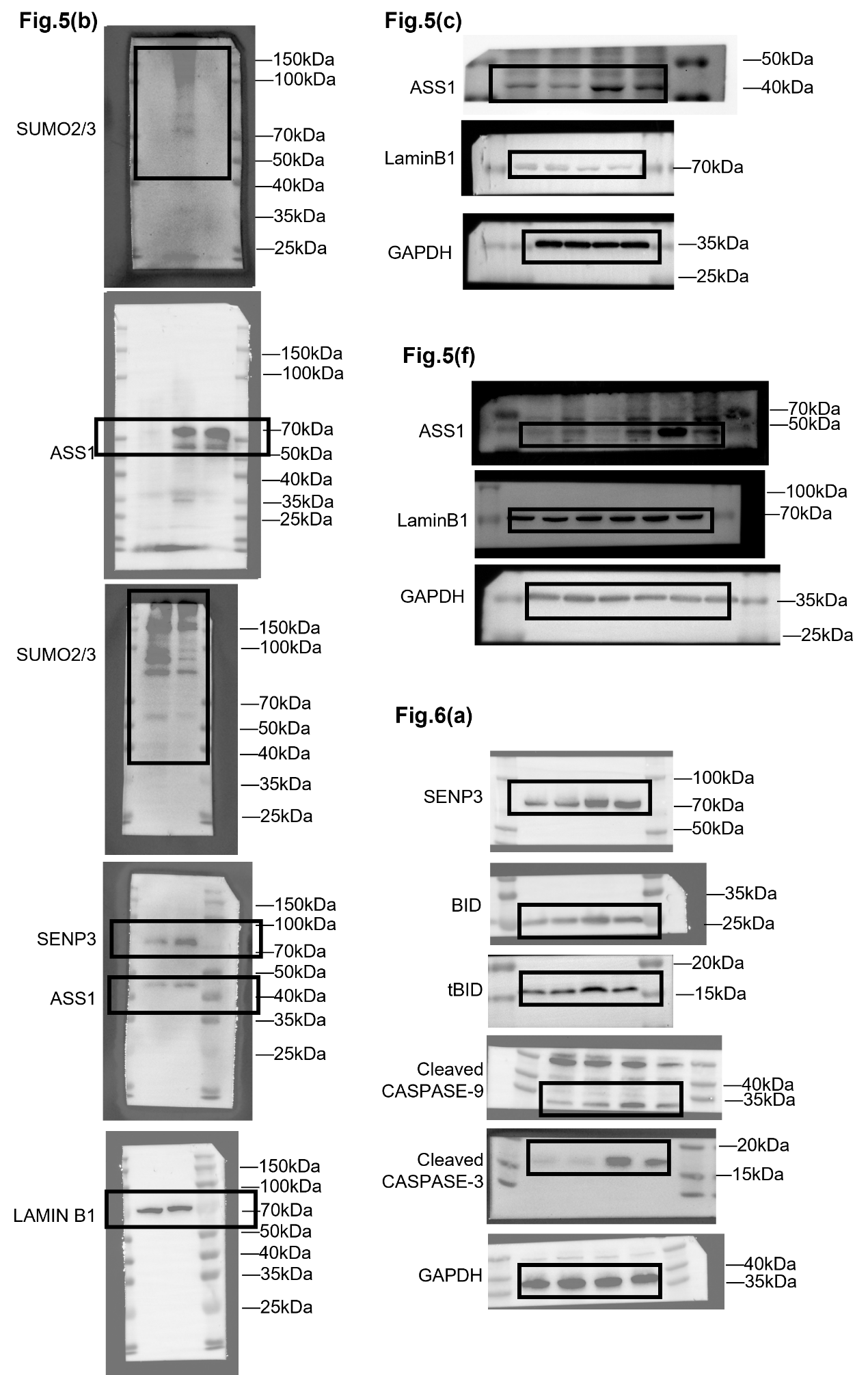

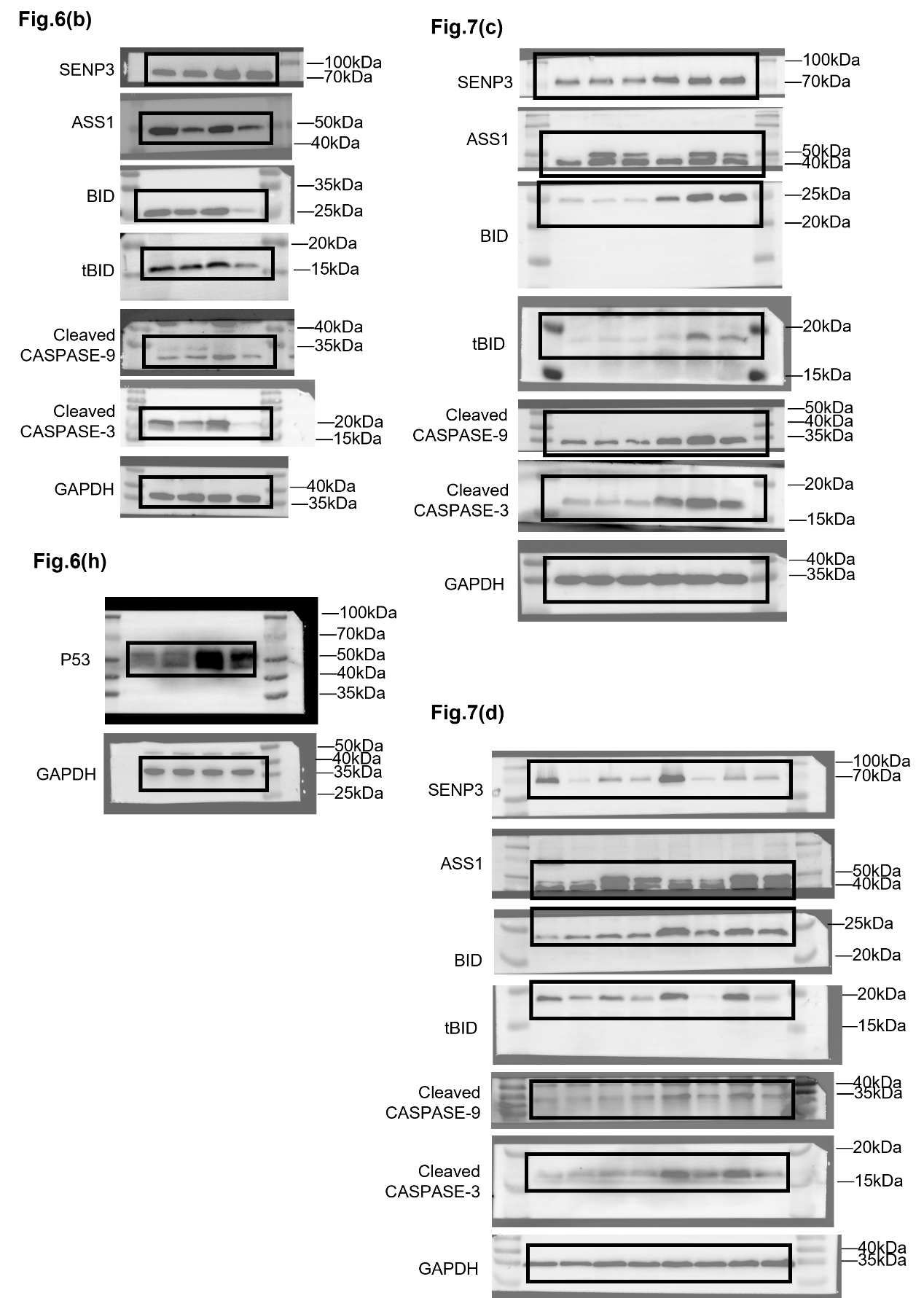

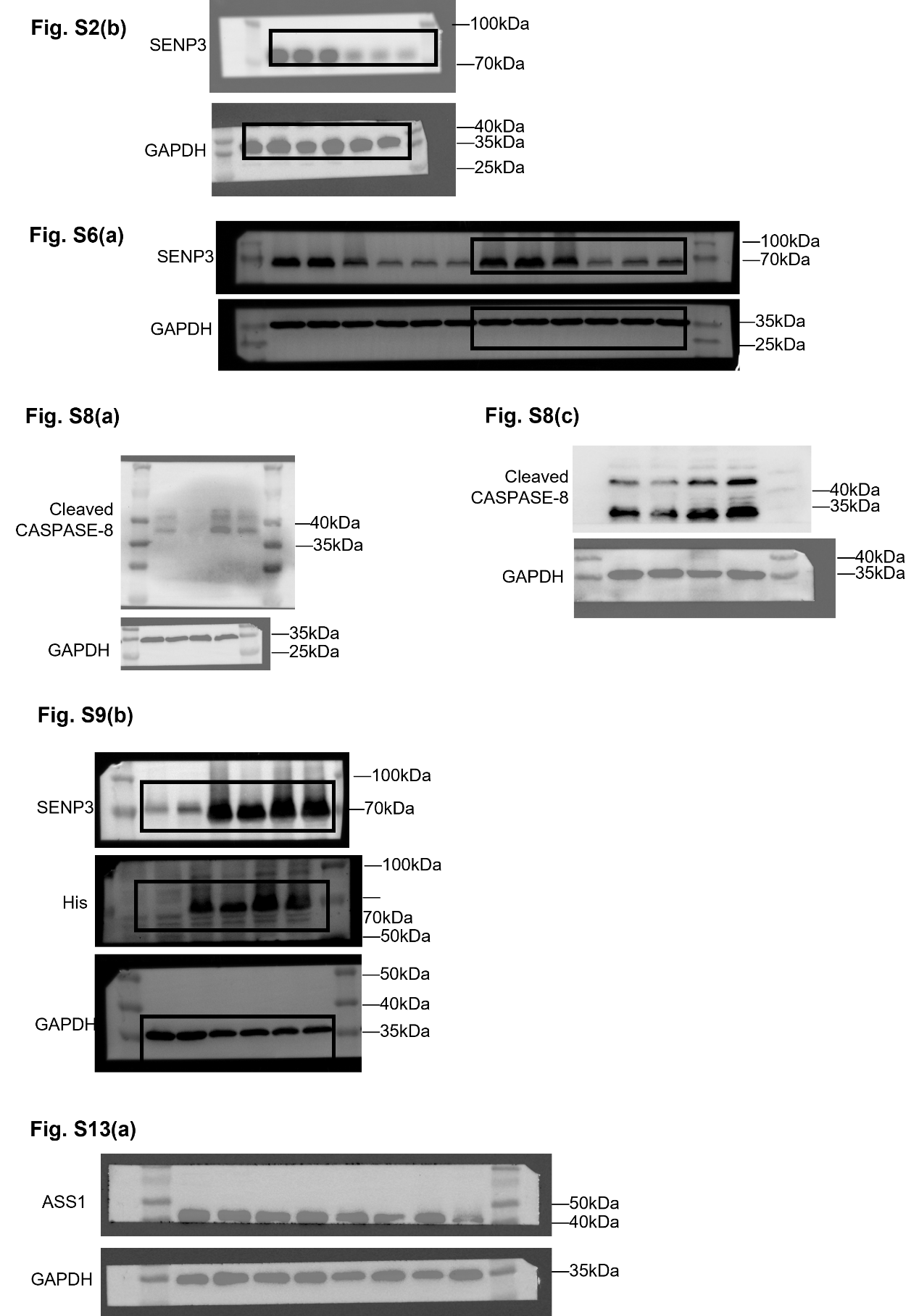

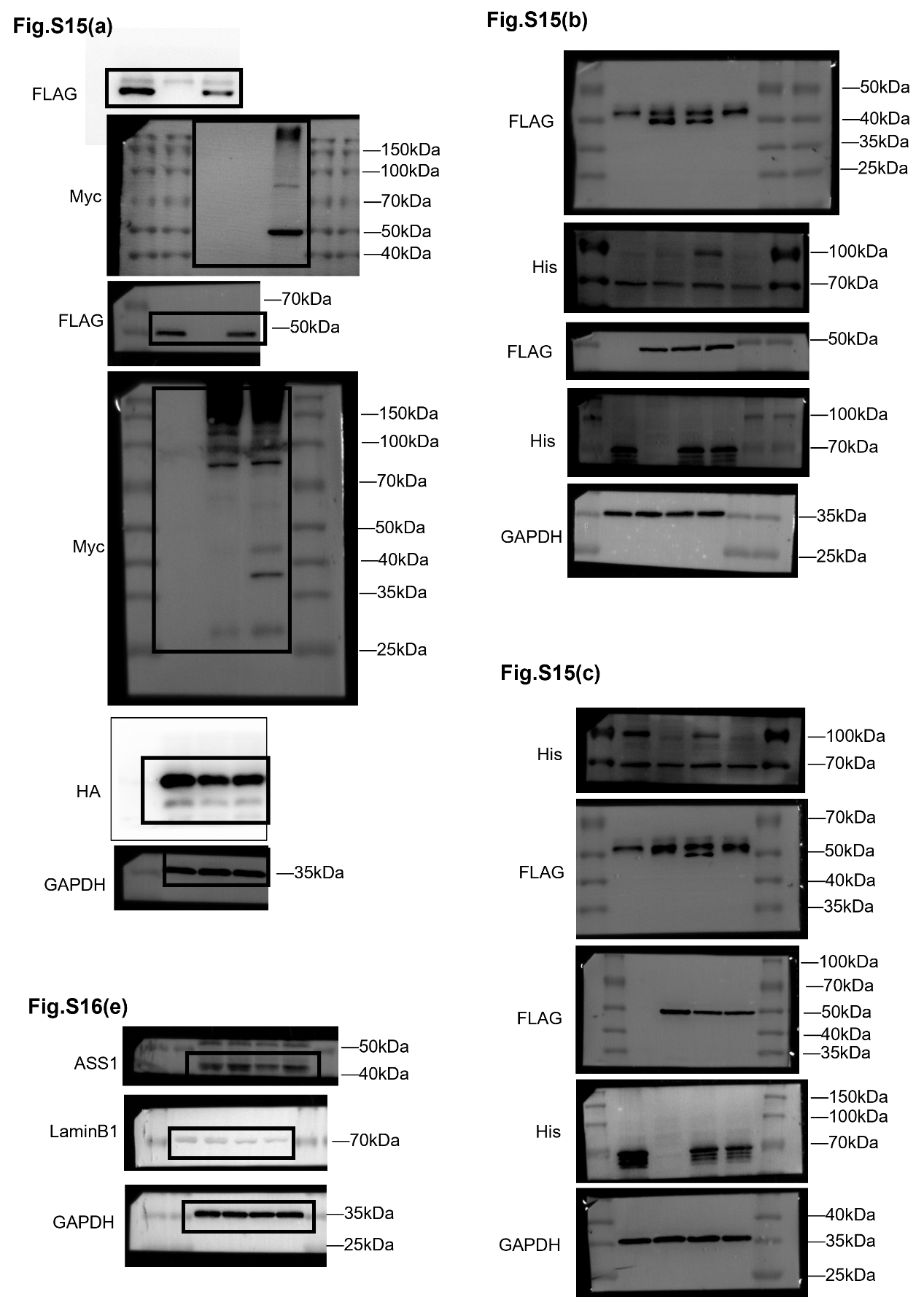


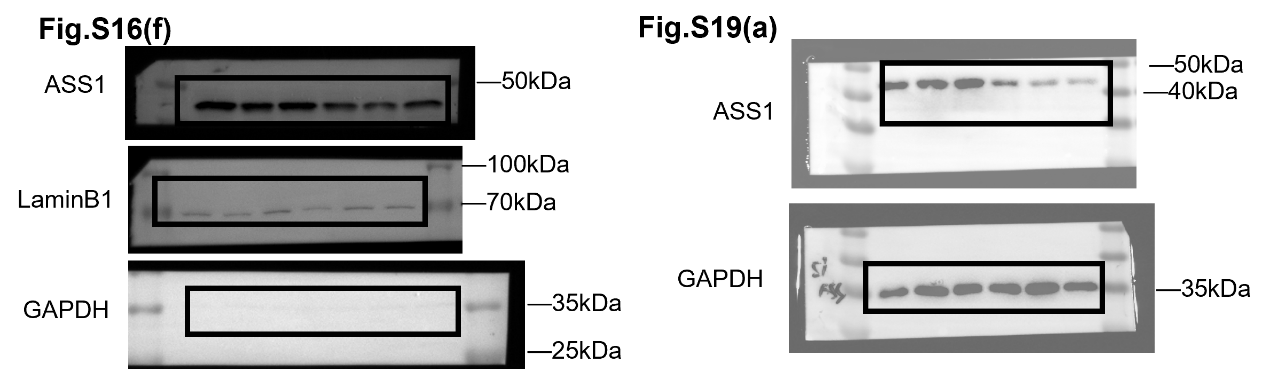

Supplement: Supplementary file 1 — supplemental material [file 41419_2025_8308_MOESM1_ESM.docx]
